# Designing High‐Rate and High‐Capacity Lithium Metal Anodes: Unveiling Critical Role of Carbon Nanotube Structure

**DOI:** 10.1002/smll.202503161

**Published:** 2025-08-07

**Authors:** Ying Zhou, Tomoko Yamagishi, Kazufumi Kobashi, Don N. Futaba, Takeo Yamada, Kenji Hata

**Affiliations:** ^1^ Nanocarbon Material Research Institute National Institute of Advanced Industrial Science and Technology 1‐1‐1 Higashi Tsukuba Ibaraki 305‐8565 Japan; ^2^ Zeon Corporation 1‐2‐1 Yako Kawasaki‐ku Kawasaki Kanagawa 210‐9507 Japan

**Keywords:** carbon nanotube, functional groups, host, Li metal anode, pore structure

## Abstract

Strategic material selection is critical for designing high‐performance energy‐storage systems. This study reveals that the intrinsic structure of carbon nanotubes (CNTs), rather than modification, plays a decisive role in achieving ultrahigh capacity and stability. Using CNT selection as a design strategy, a mesoporous Li host is developed that enables an exceptional areal capacity of 90 mAh cm^−2^, long‐term cycling stability exceeding 2000 cycles, and operation at an ultrahigh current density of 60 mA cm^−2^ in symmetric cells. Comprehensive CNT characterization reveals significant differences in diameter, wall number, length, surface chemistry, and assembly behavior, all of which critically influence the lithiophilicity, mechanical integrity, and electrolyte permeability. These factors govern the Li deposition behavior, highlighting the importance of selecting CNTs according to their intrinsic properties rather than relying on post‐processing modifications. The anode design is validated in a full Li battery cell using a commercial cathode, achieving an areal capacity of 1.5 mAh cm^−2^, an 800‐cycle lifetime, and stable operation at a current density of 1.5 mA cm^−2^, significantly surpassing conventional benchmarks. This work establishes the fundamental role of CNT structure–property relationships in Li‐metal anode design, offering a pathway for simplified processing, enhanced performance, and more reliable next‐generation Li‐metal batteries.

## Graphical abstract

This study reveals that the intrinsic structure of carbon nanotubes (CNTs), rather than chemical modifications, determines Li‐metal anode performance. With careful selection of CNTs based on their structural characteristics, a mesoporous Li host delivered an ultrahigh capacity (90 mAh cm^−2^), exceptional cycling stability (>2000 cycles), and outstanding rate capability (60 mA cm^−2^), offering a new strategy for high‐performance Li‐metal batteries.

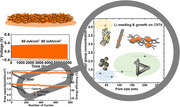

## 1 Introduction

The rapid growth of electric vehicles, portable electronics, and renewable energy storage has increased the demand for high‐performance Li‐ion batteries. Li metal is an attractive anode material because of its high specific capacity (3860 mAh g^−1^) and low electrode potential (–3.04 V vs. SHE). However, a key challenge, particularly at high current densities, is failure due to the inhomogeneous growth of Li, which leads to dendrite formation. These needlelike structures pose serious risks, causing capacity loss, reduced cycle life, and increased risks of internal short circuits and thermal runaway, thereby limiting the practical use of Li‐metal batteries.^[^ ^1^ ^]^ Achieving a high areal capacity, long cycle life, and operation at high current densities remains critical but difficult, and few studies have demonstrated success in meeting all three metrics simultaneously.^[^ ^2^ ^]^

To overcome these challenges, recent research has focused on developing 3D‐structured Li hosts using porous nanocarbon materials, such as carbon nanotubes (CNTs), graphene, and carbon fibers, to improve anode performance. These porous networks can accommodate volumetric changes, reduce the local current density, and suppress dendrite growth.^[^ ^3^ ^]^ Additionally, the incorporation of lithiophilic chemical groups, such as polar functional groups, on the carbon surfaces improves Li growth uniformity and reduces the overpotential.^[^ ^4^ ^]^ Despite these advancements, achieving a high areal capacity, long cycling stability, and operation at current densities above 10 mA cm^−2^ remains challenging owing to non‐uniform Li growth caused by high ion flux. Promising breakthroughs have been reported, such as a surface‐alloyed Li anode that achieved an areal capacity of 100 mAh cm^−2^ at 25 mA cm^−2^,^[^ ^5^ ^]^ hollow carbon nanospheres that achieved 1 mAh cm^−2^ at 64 mA cm^−2^,^[^ ^6^ ^]^ and a graphene quantum dot‐protected Li anode that maintained a capacity of 60 mAh cm^−2^ at 60 mA cm^−2^.^[^ ^7^ ^]^ Although they have achieved excellent performance, these approaches have difficulty simultaneously achieving all three criteria, particularly long cycling stability under severe conditions, as most studies demonstrated fewer than 200 cycles.^[^ ^8^ ^]^ A design that addresses all three factors: suppressing dendrite formation with a high areal capacity, long cycling stability, and operation at current densities above 10 mA cm^−2^, is needed to realize advanced high‐energy Li‐ion batteries.^[^ ^9^ ^]^

In this regard, the design of CNT‐based Li hosts is promising. Several studies have demonstrated the advantages of modifying CNTs to function as effective Li hosts, owing to their 1D morphology and ability to form 3D network structures. For example, Wang et al. demonstrated that coating molten Li onto a porous CNT film achieved high cycling stability at a current density of 40 mA cm^−2^ with a capacity of 2 mAh cm^−2^.^[^ ^10^ ^]^ N‐doping has also been shown to enhance the lithiophilic properties of CNT films, suppressing dendrite formation and enhancing cycling stability at a current density of 2 mA cm^−2^.^[^ ^11^ ^]^ Furthermore, Zhang et al. introduced an interfacial layer engineered with a lithiophilic‐to‐lithiophobic gradient using a ZnO/CNT bilayer, which improved the cycling stability over 100 cycles at 10 mA cm^−1^.^[^ ^12^ ^]^ Despite these promising developments, CNTs do not represent singular materials. In contrast to molecules, where the name refers to a specific structure, the term “CNT” encompasses a variety of graphitic, tubular nanomaterials that differ physically (in terms of diameter, length, number of walls, etc.), chemically (in terms of defects and functional‐group types and amounts), and electronically (metallic, semi‐metallic, and semiconducting). These intrinsic differences influence how CNTs assemble into macroscopic architectures, affecting properties such as bundle structure, pore size distribution, tortuosity, porosity, permeability, and mechanical durability. Therefore, selection of the appropriate type of CNT considering its inherent properties is critical, as they significantly affect the performance of the Li host and, ultimately, the overall battery performance. Previous studies addressing the role of CNT properties mainly focused on specially synthesized or chemically modified CNTs produced under precisely controlled laboratory conditions.^[^ ^10^, ^11^, ^12^, ^13^ ^]^ While such approaches allow precise tuning of the structure and surface chemistry, they do not directly reflect the challenges faced in real‐world application design, where end users must work with commercially available materials. Moreover, the deliberate combination of structural and chemical modifications in engineered CNTs often obscures the impact of intrinsic CNT attributes, making it difficult to isolate the effects of material selection. Consequently, these studies have not fully clarified how the natural variation among commercially available CNTs influences the downstream assembly behavior and performance of practical devices.

In this study, we focus on as‐received commercially available CNTs that span a broad range of intrinsic properties and have not undergone further chemical treatment or purification. This approach enhances practical relevance by relying on scalable, readily available materials that align closely with real‐world application requirements. It also allows the direct examination of naturally occurring structural features, such as pore architecture, surface area, and functional‐group distribution, without the confounding effects of post‐processing. Thus, this work advances the fundamental understanding of structure–property relationships in CNT/Li systems and helps bridge the gap between academic research and practical battery design. We fabricated a mesoporous Li host in a symmetric cell configuration that exhibited a high areal capacity (90 mAh cm^−2^) and long cycling stability (2000 cycles) at a high current density (60 mA cm^−2^). Central to this work is a comprehensive physical and chemical examination of several commercially available CNTs. Their intrinsic physical, chemical, and electronic characteristics were used to guide the design and fabrication of a mesoporous Li host with the desired structural and electrochemical properties. Extensive examination of the physical and chemical attributes of several commercially available single‐walled CNTs (SWCNTs) and multi‐walled CNTs (MWCNTs) revealed not only structural differences (average diameter, average number of walls, and length) but also differences in surface chemistry and assembly structure. The assembled CNTs exhibited significant differences in surface chemistry, bundle structure, and pore structure, which are critical for lithiophilicity, mechanical robustness, and permeability, respectively. To demonstrate the potential of this anode design, we constructed a full Li‐metal battery cell using a commercial cathode and compared its performance with literature benchmarks, highlighting the strength of this anode. Under demanding conditions of a 1C charge/discharge rate (1.5 mA cm^−2^), our Li‐SGCNT anode retained a stable capacity (1.5 mAh cm^−2^) over 800 cycles, outperforming previously reported anodes with regard to areal capacity and cycle life, operated at high current density. This study underscores the importance of understanding and leveraging the intrinsic physical and chemical differences among CNTs to simplify processing and optimize their use in energy‐storage applications. Additionally, our findings highlight how inherent CNT variations determine the downstream assembly properties, emphasizing the importance of selecting an appropriate starting material and understanding its natural assembly behavior as critical factors in designing the desired final structure.

## 2 Results and Discussion

**Figure 1:**
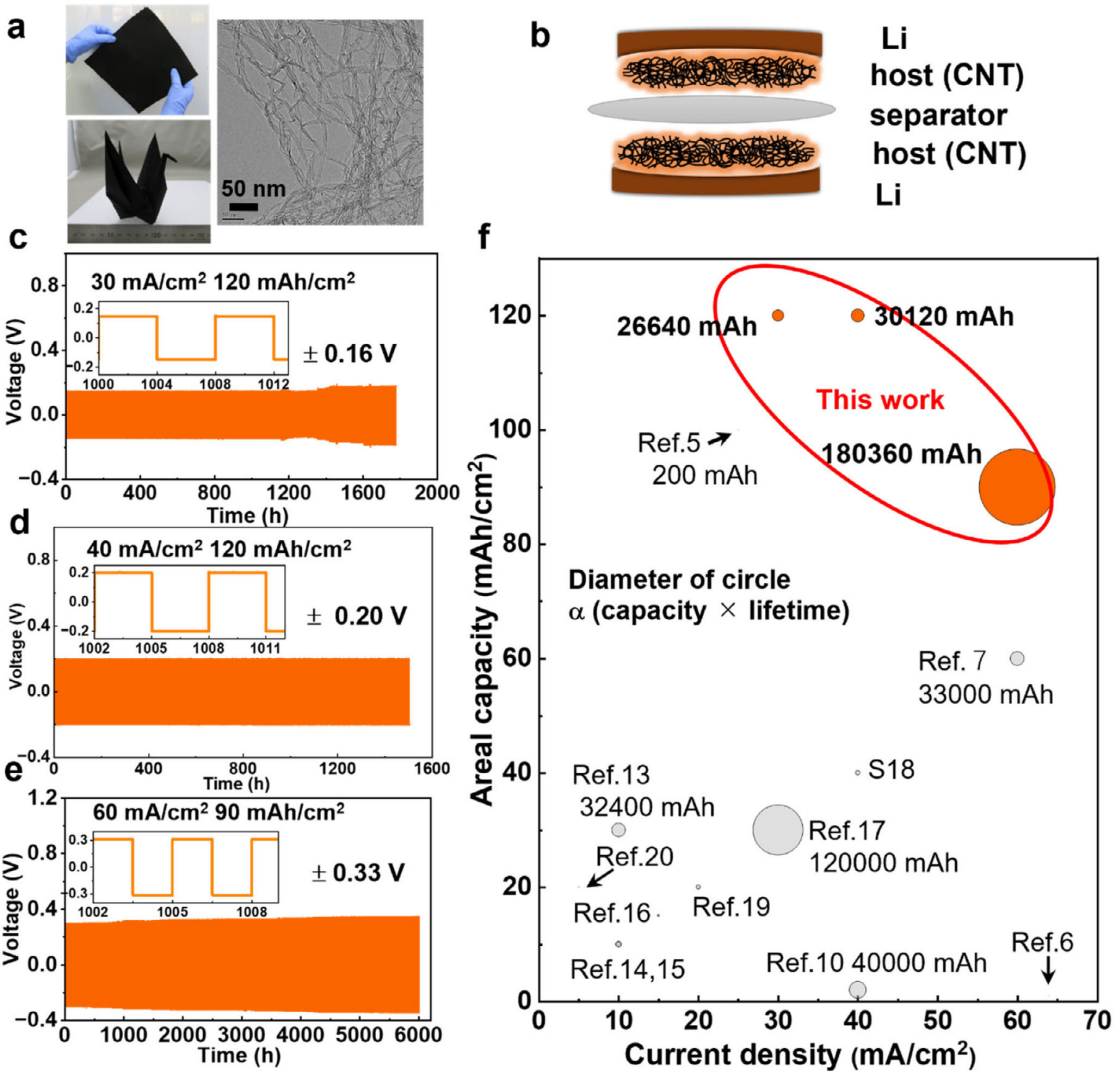
a) Photographs of SGCNT freestanding films, including an origami crane demonstration, and a corresponding transmission electron microscopy image. b) Schematic of symmetric cells used for evaluating anode performance. Galvanostatic cycling profiles of symmetric cells operated at c) 30 mA cm^−2^ and 120 mAh cm^−2^, d) 40 mA cm^−2^ and 120 mAh cm^−2^, and e) 60 mA cm^−2^ and 90 mAh cm^−2^. f) Comparison of the current densities and areal capacities of symmetric cells with the SGCNT hosts and previously reported Li‐metal anodes prepared using various methods (Table S1, Supporting Information). The diameter of each circle represents the cumulative plating capacity (plating capacity × cycle number), shown for only reports exceeding 20000 mAh cm^−2^.

First, we demonstrated that a CNT‐based Li‐metal anode can achieve exceptional performance using as‐received SWCNTs without additional treatment. We used a symmetric cell configuration with a binder‐free SWCNT film as the Li host. For the SWCNT films, we employed CNTs prepared via water‐assisted chemical vapor deposition, i.e., “super‐growth carbon nanotubes (SGCNTs),” as the Li host owing to their high carbon purity, exceptionally high purity, and large specific surface area. Importantly, the SGCNTs were used in their as‐received state without chemical modification, purification, or specialized assembly. Freestanding, binder‐free SGCNT films ≈100 µm thick with a loading mass of 2 mg cm^−2^ were fabricated through standard filtration of a surfactant‐free aqueous dispersion. The robustness of the film assembly was demonstrated by its ability to fold into an origami crane (**Figure** **1** **a**). Symmetric cells comprising two identical SGCNT (100‐µm thick) and Li (750‐µm thick) electrodes separated by a polyethylene separator were fabricated (Figure 1b). The galvanostatic plating and stripping of Li occurred repeatedly in an electrolyte solution of 1 M LiPF_6_ in EC/DMC (1:1, vol) with 5 vol% FEC. We evaluated the cell performance using symmetric cells because they provide a clear understanding of the Li plating/stripping processes and electrode stability. This configuration isolated the performance of the Li‐metal anode and CNT host materials without interference from the counter electrode, facilitating analysis of the overpotential, uniformity of Li deposition, and dendrite suppression, which are crucial for assessing long‐term cycling stability and rate capability.

Prior to testing, the symmetric cells underwent initial treatment at a current density of 0.5 mA cm^−2^ and areal capacities ranging from 5 to 20 mAh cm^−2^ several times to eliminate reactive functional groups and establish a uniform solid electrolyte interphase (SEI) layer on the CNT surfaces. The symmetric cells were then cycled at current densities of 30, 40, and 60 mA cm^−2^ with areal capacities of 120, 120, and 90 mAh cm^−2^, respectively, as shown in Figure 1c–e. The cells with the SGCNT host exhibited exceptional performance, with stable voltage profiles and minimal overpotentials across various conditions, demonstrating the tolerance of the cells to extreme conditions for Li plating/stripping. The voltage profiles remained stable throughout cycling, with measured plating/stripping overpotentials of ≈0.16, 0.20, and 0.33 V for 30, 40, and 60 mA cm^−2^, respectively. These values indicate the energies needed to drive the plating and stripping reactions at these current densities. Lower overpotentials reflect more efficient electrochemical processes and reduced resistance, which are critical for maintaining stable and uniform Li deposition, particularly at high current densities. Remarkably, even at an ultrahigh current density (60 mA cm^−2^) and areal capacity (90 mAh cm^−2^), the Li‐SGCNT anodes maintained their long‐term stability, lasting >6000 h (2000 cycles). Figure 1f compares the performance of the best Li‐metal anodes reported in recent literature,^[^ ^5^, ^6^, ^7^, ^10^, ^14^, ^15^, ^16^, ^17^, ^18^, ^19^, ^20^, ^21^ ^]^ highlighting that the use of SGCNT results in not only high current density and areal capacity but also exceptional cyclability, with a cumulative plating/stripping capacity exceeding 180 000 mAh cm^−2^.

**Figure 2:**
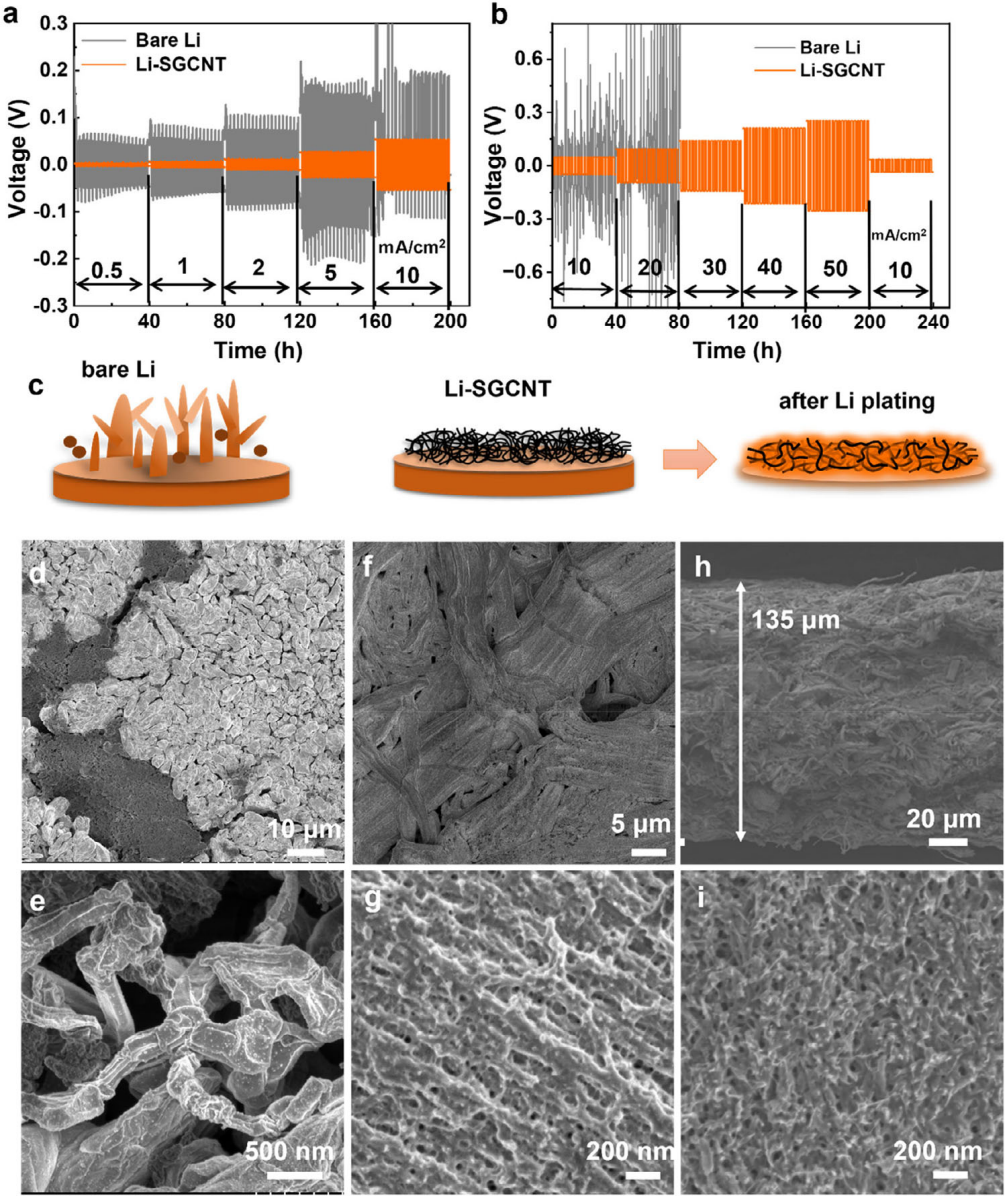
Galvanostatic cycling profiles of Li‐SGCNT and bare Li symmetric cells operated at various current densities and capacities: a) 0.5–10 mA cm^−2^ and b) 10–50 mA cm^−2^. c) Schematics illustrating dendrite suppression by SGCNT films. d,e) SEM images of bare Li after plating/stripping cycles, as shown in (a). f,g) SEM images and h,i) cross‐sectional SEM images of Li‐SGCNTs after plating/stripping cycles as shown in (a).

The Li stripping and plating behavior validated the superior performance of the Li‐SGCNT‐based symmetric cells compared with bare Li electrodes. Li‐SGCNT cells exhibited stable cycling with low overpotentials across a wide range of current densities and areal capacities, as shown in **Figure** **2a,b**. Specifically, Li‐SGCNT cells exhibited low overpotentials of 3 and 27 mV at current densities of 0.5 and 5 mA cm^−2^, respectively. Even at higher current densities of 10–50 mA cm^−2^, the voltage profiles remained flat with a near‐linear increase as the current density increased, indicating efficient electron and ion transport as well as stable plating/stripping behavior on the SGCNTs. In comparison, bare Li symmetric cells exhibited significantly higher overpotentials of ≈60 and 200 mV at current densities of 0.5 and 5 mA cm^−2^, respectively. They also had unstable voltage profiles, particularly at current densities exceeding 5 mA cm^−2^, suggesting poor Li plating/stripping behavior with increased polarization.

In this study, thick Li‐metal foils (≈750 µm) with a theoretical capacity of ≈145 mAh cm^−2^ were used. This arrangement cannot fully replicate practical Li‐metal anodes, which typically exhibit limited Li availability, and thus does not rigorously represent real‐world performance. However, our approach eliminates limitations related to Li depletion, allowing the isolation and detailed evaluation of the intrinsic stability of the Li plating and stripping processes influenced by CNT structures. The results clearly demonstrate that the SGCNT films significantly suppress dendrite formation and facilitate uniform Li deposition, even at high areal capacities under aggressive cycling conditions. ≈60% and 83% of the Li were effectively utilized at areal capacities of 60 and 40 mAh cm^−2^, respectively, during extended cycling. Moreover, as shown in Figure S1 (Supporting Information), the use of 200‐µm‐thick Li foils enabled stable cycling for high areal capacities of 10 and 20 mAh cm^−2^ at 10 and 20 mA cm^−2^, respectively. These results further highlight the potential of SGCNTs as Li hosts.

In addition to the observed electrochemical advantages, morphological analysis after cycling highlighted the ability of the Li‐SGCNT system to suppress inhomogeneous Li nucleation and dendrite formation (Figure 2c). The cycled bare Li anodes exhibited increased surface roughness and irregularly branched Li deposits (Figure 2d,e), whereas the Li‐SGCNT surface remained smooth and uniform (Figure 2f,g). The cross‐sectional SEM images after cycling revealed that the SGCNT films expanded to accommodate Li deposition without the formation of dendrites or dead Li, maintaining a dense and stable structure (Figure 2h,i). These results demonstrate that SGCNT films can serve as exceptional Li hosts in symmetric cells; however, the specific structural and chemical attributes responsible for this performance remain unclear. To address this issue, we compared the intrinsic properties of the SGCNTs with those of commercially available CNTs.

As mentioned previously, CNTs differ in almost every aspect, including physical properties (e.g., average diameter, average number of walls, and length) and surface reactivity (e.g., distribution, concentration, and type of surface functional groups), which stems from their distinct methods of synthesis, storage, and processing. We demonstrated the significant impact of these factors on the assembly structure and plating/stripping behavior. For this purpose, we examined four additional commercial CNTs: two SWCNTs (eDIPS and Tuball) and two MWCNTs (JEIO and K‐Nanos). The eDIPS and Tuball CNTs had average diameters of ≈1.5 nm and bundle lengths of tens of micrometers. The JEIO and K‐Nanos CNTs had average diameters of 9.1 and 4.3 nm, average wall numbers of 6.2 and 2.4, and average bundle lengths of tens and hundreds of micrometers, respectively.^[^ ^22^ ^]^ For each variety, flexible, binder‐free, freestanding CNT films (Table S2, Supporting Information) with areal densities of ≈2 mg cm^−2^ were prepared, and their Li plating/stripping behavior was evaluated using symmetric cells. Freestanding films, serving as potential Li hosts, not only facilitate the preparation of the Li/CNT bilayer configuration but also offer sufficient mechanical strength to accommodate Li plating.

The symmetric cell performance of commercial CNTs, including eDIPS, Tuball, JEIO, and K‐Nanos, exhibited higher and more unstable overpotentials during Li stripping and plating, particularly at current densities above 2 mA cm^−2^ (Figure S2, Supporting Information), compared with Li‐SGCNT (Figure 2). Figure S3 (Supporting Information) shows the time‐resolved electrochemical impedance spectroscopy (EIS) spectra after galvanostatic cycling at various current densities. While bare Li exhibited a significant increase in impedance after cycling, SGCNTs maintained stable impedance spectra across all current densities, suggesting the formation of a stable SEI. Similar to bare Li, Li‐JEIO exhibited an increased impedance, likely due to continuous SEI growth. In contrast, Li‐eDIPS demonstrated a marked decrease in impedance after cycling at 5 mA cm^−2^, possibly indicating a short‐circuit event.

**Figure 3:**
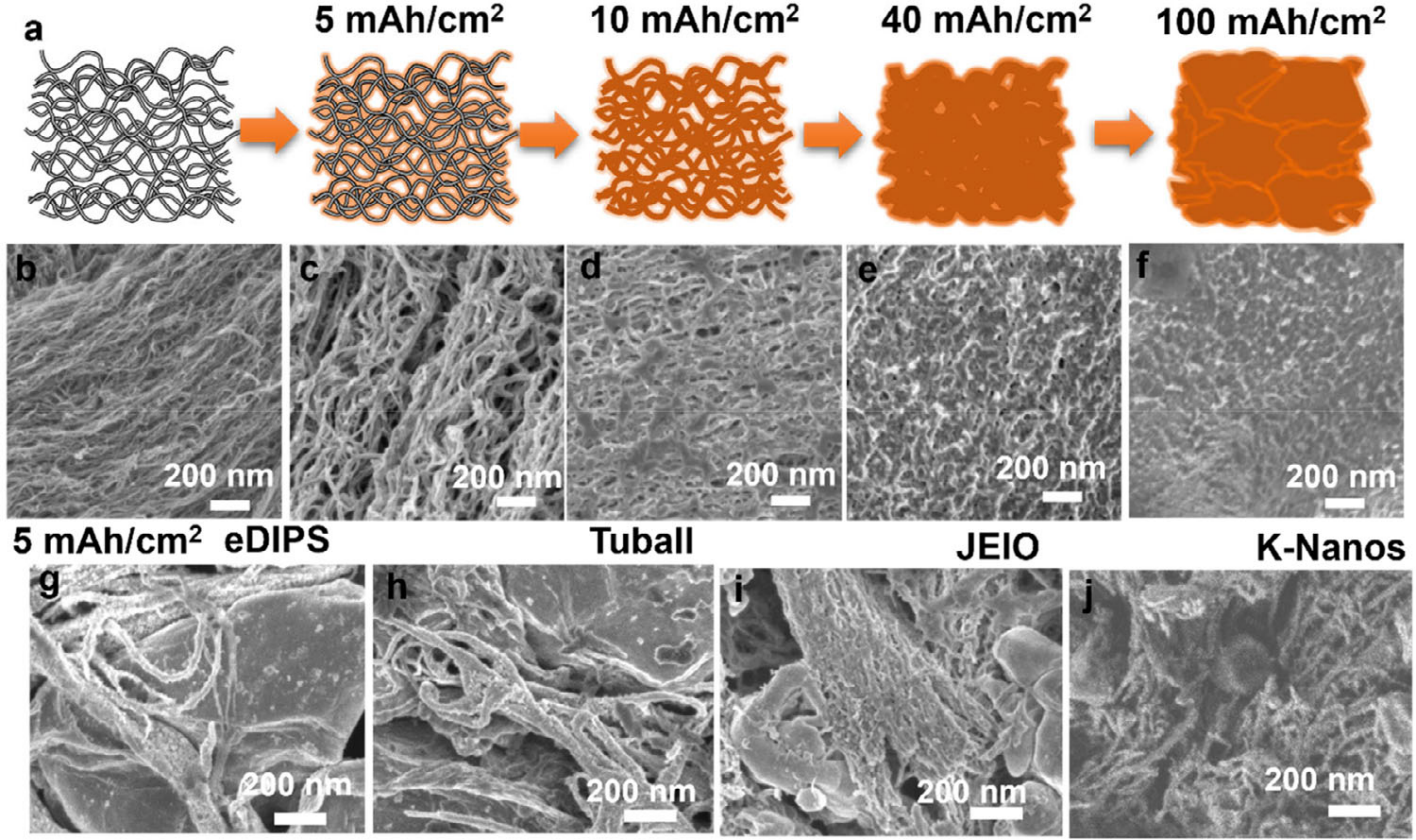
a) Schematics and b–f) corresponding SEM images capturing the progression of SGCNT films before and after electrochemical Li plating, ranging from 5 to 100 mAh cm^−2^ at a current density of 1 mA cm^−2^. SEM images of g) eDPIS, h) Tuball, i) JEIO, and j) K‐Nanos films after electrochemical Li plating at 5 mAh cm^−2^ are also presented.

To investigate the morphological evolution during Li plating, we examined the SGCNT films and compared them with other CNT types (eDIPS, Tuball, JEIO, and K‐Nanos) across the plating range of 5–100 mAh cm^−2^ (**Figure** **3** **a–f**). Li plating on the SGCNT films caused significant structural modifications. At 5 mAh cm^−2^, the CNT bundles expanded, as stated previously, forming uniform layers consistent with the SEI formation and increasing the pore size to ≈100 nm (Figure 3c). As the plating rate increased to 40 mAh cm^−2^, the pores filled uniformly, resulting in a flat and smooth surface (Figure 3e). At 100 mAh cm^−2^, a dense, flat layer formed, with cross‐sectional SEM images (Figure S5, Supporting Information) showing the thickness increasing from 100 to >500 µm. Moreover, the SGCNT films expanded approximately threefold (≈300 µm) to accommodate the uniform Li precipitation, which filled the pores within the bundles rather than solely along the periphery of the CNT bundles. This observation is consistent with the expanded pore structure of the SGCNTs, as shown in Figure 3c. Furthermore, near the separator, denser Li deposits formed continuous layers with no significant dendrites or dead‐Li accumulation. These results suggest that higher areal capacities can be achieved by supplying sufficient Li. Additionally, we evaluated SGCNT films with various thicknesses and found that optimal performance was achieved in the range of 50–150 µm, with similar overpotentials and capacities at current densities below 10 mA cm^−2^.

In comparison, the other CNT types exhibited less favorable morphological changes and inconsistent Li growth. The eDIPS and Tuball films formed large precipitates (>200 nm) during the initial plating, which developed into micro‐sized crystalline masses with further plating, as shown in Figure 3g,h. The JEIO and K‐Nanos films exhibited smaller, randomly distributed precipitates (Figure 3i,j) with continuous plating, resulting in isolated dead‐Li deposits covering their surfaces (Figure S4, Supporting Information). These variations in the Li growth morphology across different CNTs agree well with the EIS results (Figure S3, Supporting Information). Dendritic or dead Li promotes the formation and evolution of an unstable SEI, increasing both ion transport and interfacial resistance, as reflected by the increasing EIS impedance over time. Notably, the significant crystalline growth observed on the eDIPS CNTs damaged the separator, resulting in a short circuit and a marked decrease in the EIS impedance (Figure S3, Supporting Information). The Li‐SGCNT electrode promoted the formation of a stable SEI, as evidenced by stable EIS responses and consistent Li stripping/plating behavior. Moreover, the MWCNTs did not exhibit significant volumetric expansion, likely because of their mechanical stiffness, which restricted such changes. These comparative results underscore the critical importance of a stable CNT assembly structure, as demonstrated by the SGCNTs. Furthermore, the findings highlight how the distinct assembly structures (void formations, CNT–CNT packing) and surface chemistries of various CNTs govern the Li growth morphology. To further investigate these effects, we examined the pore structure and surface functional groups of a set of CNTs.

**Figure 4:**
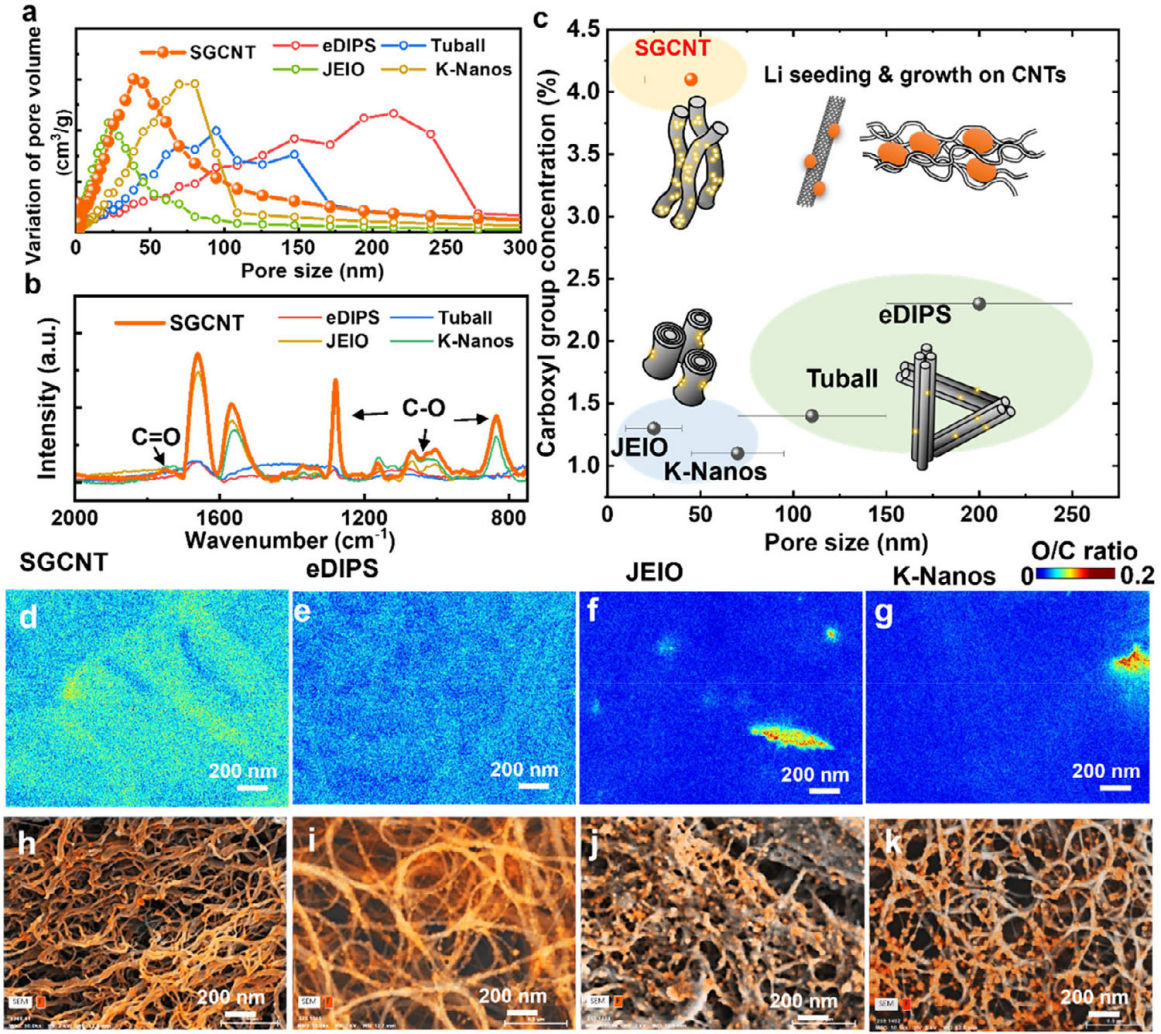
Effects of functional groups and pore structures in CNT films on Li growth. a) Pore size distribution of CNT films derived from N_2_ adsorption isotherms. b) FTIR spectra comparing functional groups in SGCNT films with those in other commercial CNTs. c) Comparison of structural characteristics of CNTs that affect Li seeding and growth. Elemental mapping images illustrating the O/C intensity ratio for d) SGCNT, e) eDIPS, f) JEIO, and g) K‐Nanos. SEM‐EDS images combined with F mapping after a single cycle of electrochemical Li plating/stripping at 0.2 mA cm^−2^ and 2 mAh cm^−2^ for h) SGCNTs, i) eDIPS, j) JEIO, and k) K‐Nanos.

Differences in pore structure among the CNTs are expected owing to variations in diameter, length, crystallinity, and impurities, all of which influence the intimacy of the CNT–CNT contacts. The pore structure directly supports accessibility to the CNT surface, which governs the Li‐ion transport, areal capacity, current density, and lifetime (if structurally stable). For this purpose, the pore size distribution for each CNT film was determined using the Barrett–Joyner–Halenda (BJH) method from the N_2_ adsorption isotherms, which revealed distinct characteristics, as shown in **Figure** **4** **a** and Figures S6 and S7 (Supporting Information). The CNTs exhibited a range of pore sizes that significantly influenced their performance during Li plating. The SGCNTs exhibited a mesoscopic pore structure ranging from 30 to 60 nm, providing an ideal environment for uniform Li precipitation. After plating at 10 mAh cm^−2^, the SGCNT bundles were uniformly filled with nanostructured precipitates (≈50 nm in diameter), leading to stable and homogeneous Li growth. Moreover, larger pore structures were observed for both the eDIPS and Tuball SWCNTs (>200 nm). We inferred that the large pore size reduced the available surface area for Li deposition, leading to the formation of precipitates after only 5 mAh cm^−2^ plating (Figure 3g,h). However, the pore size does not fully explain the MWCNT results. Notably, JEIO and K‐Nanos, which exhibited different pore sizes (20 and 80 nm, respectively), did not guide the uniform growth of Li within their pore structures. For K‐Nanos, Li tended to be deposited on the bundle surfaces rather than in the pores, whereas for JEIO, the pore structures were filled with an SEI layer, preventing the uniform guidance of Li growth (Figure 3i,j).

The types and amounts of functional groups present on CNTs are important factors affecting their use as Li host materials. Similar to defect‐free graphene,^[^ ^23^ ^]^ CNTs are chemically inert, and the type and quantity of surface‐bound functional groups (i.e., stable reaction sites) have been reported to be beneficial for longer battery cycling because of their ability to promote the formation of a stable and uniform SEI and thus uniform Li plating and stripping across the surfaces.^[^ ^24^ ^]^ All CNTs inherently contain crystalline defects that serve as sites for functional groups. Owing to fundamental variations in the radius of curvature and synthesis methods, substantial differences in surface chemistry are expected among CNT varieties. However, these differences have rarely been discussed. Our spectroscopic analysis using Fourier transform infrared (FTIR) spectroscopy revealed differences in the functional groups of the CNTs (Figure 4b), which may correspond to the observed differences in the Li stripping and plating behavior.^[^ ^25^ ^]^ SGCNT, K‐Nanos, and JEIO exhibited several strong vibrational peaks corresponding to carboxyl and hydroxyl groups, whereas for eDIPS and Tuball, these peaks were minimal or absent. The TPD‐MS results indicated that the SGCNTs had the highest concentration of carboxyl groups at 4.1% (Figures S8 and S9, Supporting Information), equating to ≈2 functional groups per 100 carbon atoms, which favors uniform Li nucleation. Raman spectroscopy indicated lower ratios of graphitic‐to‐disorder peaks (G/D ratios), indicative of more structural defects, for SGCNTs, K‐Nanos, and JEIO (≈1) than for eDIPS and Tuball (>60) (Figure S10, Supporting Information). Notably, the presence of structural defects generally corresponds to sites for functional‐group bonding. Figure 4c summarizes the structural characteristics of these CNTs, which influence their Li plating/stripping behaviors. The SGCNTs have a high concentration of carboxyl groups and an optimal pore size distribution. The functional‐group concentration in eDIPS may be overestimated, as the spectroscopic analysis (Figure 4a; Figures S10 and S11, Supporting Information) indicated that eDIPS had high crystallinity with minimal oxygen‐containing defects. This was supported by scanning electron microscopy‐energy‐dispersive X‐ray spectroscopy (SEM‐EDX) elemental mapping (Figure 4d, which revealed a homogeneous and high concentration of oxygen throughout the SGCNT network, whereas eDIPS and Tuball exhibited approximately one‐fifth of the oxygen levels owing to their higher crystallinity. In contrast, the O/C distributions for K‐Nanos and JEIO were not uniform, suggesting that their functional groups were inconsistently distributed, likely because of impurities such as catalyst residues and/or amorphous carbon. We suspect that, as typical mass‐produced MWCNTs, K‐Nanos and JEIO lack precise structural control and often contain significant impurities and non‐uniform structural defects, which compromise their functionalization consistency and limit their effectiveness as Li hosts. Functional groups on the CNT surface can enhance the interfacial compatibility with electrolyte by increasing the local polarity, thereby improving interfacial accessibility and facilitating ion/charge transport. This enhanced lithiophilicity promotes uniform Li nucleation and growth, contributing to the formation of a homogeneous and stable SEI. Functional groups may promote the decomposition of electrolytes to generate LiF, which is a key component of robust SEI layers.^[^ ^3^, ^7^ ^]^ The inherent presence of functional groups, particularly in SGCNTs, enhances uniform Li nucleation and growth by promoting the formation of a continuous LiF layer for SEI stabilization. FTIR analysis revealed that all CNTs possessed similar types of functional groups, suggesting that they underwent comparable reactions to form the SEI, including the generation of inorganic LiF and other species.^[^ ^24^ ^]^ However, the concentration and spatial distribution of these functional groups can significantly influence the morphology and uniformity of the SEI. Because F in the SEI was present exclusively as LiF, mapping the F distribution provides insight into the LiF morphology. Although SEM‐EDS has a limited chemical resolution, it provides valuable spatial information at low accelerating voltages, making it well‐suited for visualizing inorganic SEI components such as LiF.^[^ ^26^ ^]^ As shown in Figure 4h–k, the F‐mapping SEM‐EDS results of the CNT films after Li plating (2 mAh cm^−2^, ≈1000 mAh g^−1^) revealed a continuous, homogeneous distribution of F on the SGCNT bundles. This implies that the inherent functional groups of the SGCNTs create a favorable environment for the formation and uniform distribution of LiF, which is a key component of a stable SEI.^[^ ^27^ ^]^ A well‐distributed LiF layer enables more uniform Li plating/stripping, reducing the localized hotspots that could lead to dendrite growth. By maintaining an intact and well‐regulated SEI, the F‐mapping results confirmed that the functional groups on the SGCNTs promoted uniform Li nucleation and growth, enhancing the plating/stripping behavior for long‐term cycling stability. In contrast, although eDIPS and Tuball exhibited a generally uniform F distribution, small F‐rich “islands” formed on the CNT bundles. Moreover, Figure 4j,k show the formation of isolated LiF particles on JEIO and K‐nanos, respectively, indicating that LiF formation in these CNTs was significantly affected by their non‐uniform distributions or fewer functional groups. A comparative analysis of the F distribution across different CNTs highlighted the importance of CNT surface chemistry in facilitating the formation of a continuous LiF layer, which promotes a stable SEI and uniform Li plating. These results are consistent with the stable impedance spectra observed for the Li‐SGCNTs, as shown in Figure S3 (Supporting Information).

Moreover, both experimental and theoretical studies have demonstrated that mesoscopic structures with lithiophilic surfaces can homogenize the electric field and ion flux distribution within Li‐metal anodes, thereby suppressing dendrite formation and enabling stable cycling performance.^[^ ^3^, ^4^, ^7^, ^10^ ^]^ Consistent with these findings, our results clarify that the superior performance of SGCNT films arises from a unique combination of favorable pore structures, which provide access to a large surface area and a high concentration of functional groups that promote homogeneous Li growth. These features enhance electrolyte accessibility, facilitate ion transport, and reduce the local current density during Li plating, all of which contribute to significantly lower overpotentials, as shown in Figure 1c–e. This combination allows the SGCNT films to serve as highly effective hosts for high‐capacity Li‐metal anodes, even at elevated current densities, by maintaining an adaptable structure, suppressing dendrite formation, and supporting uniform Li plating and stripping. In comparison, small‐diameter, relatively short, and highly crystalline SWCNTs tended to assemble into bundles with closely packed contacts, a broader range of pore sizes, and fewer functional groups. Under these circumstances, Li deposition was limited to the outer surfaces of the CNT bundles, restricting accessibility and uniform growth. Although the larger diameters of MWCNTs provide relatively favorable pore structures, their lithiophobic surfaces, limited surface area, and uneven distribution of functional groups hinder electrolyte infiltration and ion transport within the assembly, resulting in non‐uniform Li nucleation and growth. Additionally, commercial CNTs can exhibit batch‐to‐batch variations in key parameters that may affect their electrochemical behavior. Nevertheless, our results demonstrate that the intrinsic structural characteristics, including the wall number, diameter, crystallinity, porosity, and native functional groups, significantly affect the performance of Li‐metal anodes. By systematically selecting a diverse set of commercially available CNTs that encompass a broad spectrum of these properties, generalizable structure–property relationships that are not limited to specific suppliers or product lines can be obtained. These insights provide valuable guidance for the design and optimization of CNT‐based materials for high‐performance Li batteries.

Thus far, our results have indicated that the as‐received CNTs possess assembly features appropriate for high‐performance Li batteries in a symmetric cell configuration. To demonstrate the potential of our Li‐metal anode, we fabricated a full battery cell that exhibited exceptional performance, i.e., high areal capacity, long cycling stability, and operation at high current densities. Full 2032‐type coin cells were constructed using an SGCNT‐supported 200‐µm‐thick Li anode, a commercial LiFePO_4_ (LFP) cathode with an areal capacity of 1.5 mAh cm^−2^, and a polyethylene separator. A total of 2032‐type coin cells with an SGCNT‐Li//LFP structure and a diameter of 1.6 cm were assembled. For comparison, Li//LFP cells identical to the bare Li anodes were fabricated.

**Figure 5:**
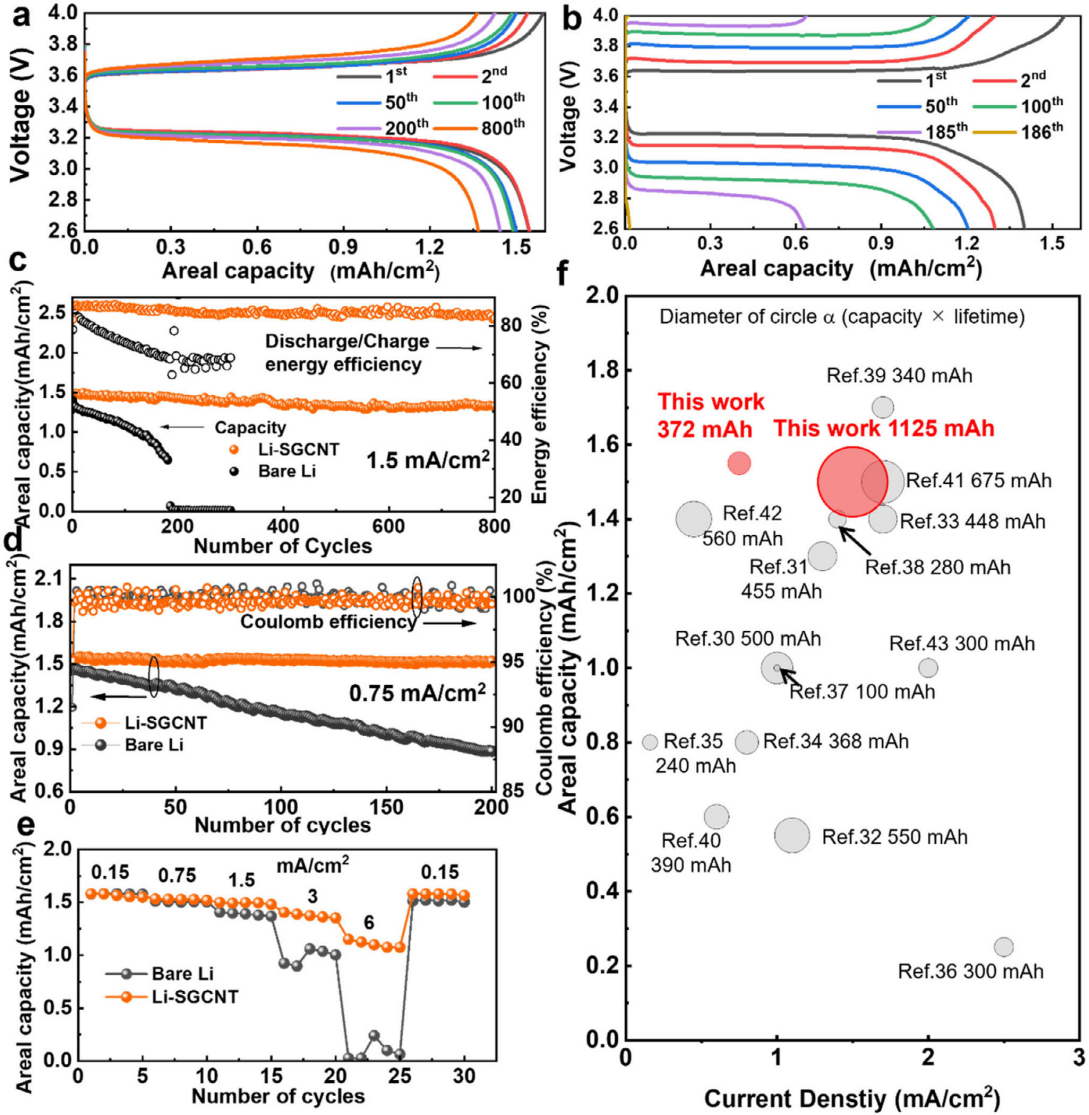
Galvanostatic voltage profiles of a) Li‐SGCNT//LFP and b) Li//LFP full cells with a cathode loading of 1.5 mAh cm^−2^ at 1.5 mA cm^−2^. Long‐term cycling performance at current densities of c) 1.5 mA cm^−2^ and d) 0.75 mA cm^−2^. Energy efficiency was determined as the ratio of discharging (discharge capacity × voltage) to charging (charge capacity × voltage) energies. e) Rate performance of full cells. f) Performance comparison of liquid‐type batteries utilizing Li‐metal anodes. The current densities and cumulative areal capacities from previous reports are presented (Table S3, Supporting Information).

Galvanostatic charge/discharge voltage testing of the Li‐SGCNT and Li cells at a current density of 1.5 mA cm^−2^ (rate of ≈1C, achieving full discharge or charge in 1 h) revealed charging curves similar to those for the 1st cycle (**Figure** **5** **a**). A current density of 1.5 mA cm^−2^ was selected to evaluate the battery performance, efficiency, and stability under a moderate load, reflecting typical real‐world usage scenarios. The Li‐SGCNT//LFP full cell exhibited a high discharge capacity of 1.51 mAh cm^−2^ and a high Coulombic efficiency of 94.3%, indicating the absence of side reactions. In contrast, the Li//LFP cell using bare Li exhibited a significant reduction in discharge capacity to 1.40 mAh cm^−2^ and a Coulombic efficiency of 91.4% (Figure 5b). The relatively high Coulombic efficiency observed for Li‐SGCNT//LFP, together with the low overpotentials during cycling, indicates enhanced ion transport and improved lithiophilicity for Li deposition.^[^ ^28^, ^29^ ^]^ This combination resulted in a noticeable increase in capacity from the first cycle, attributed to the lower polarization potential (overpotential) during the charge and discharge processes of the Li‐SGCNT anode. In the cycling evaluation, the Li‐SGCNT//LFP full cell exhibited an excellent long‐term cycling performance. After 800 charge/discharge cycles at 1.5 mA cm^−2^, the capacity remained at 90%, with an average Coulombic efficiency above 99.9%. In addition, the energy efficiency (energy ratio of discharge to charge) remained as high as 82% (Figure 5c), highlighting the cycling stability and low polarization potential of the Li‐SGCNT//LFP full cell. In contrast, the Li//LFP cell exhibited sharp reductions in capacity and energy efficiency. After 150 cycles, the discharge capacity and energy efficiency of Li//LFP cell dropped to 0.88 mAh cm^−2^ and 70%, respectively. The Li//LFP cell could not be charged below 4.0 V, because of the significantly increased polarization potential after 186 cycles, as indicated by the increased gap between the charge and discharge voltage curves (Figure 5b). These findings are consistent with the EIS results shown in Figure S12 (Supporting Information). Long‐term cycling tests at a current density of 0.75 mA cm^−2^ (≈0.5C) (Figure 5d) indicated that after 200 cycles, the Li‐SGCNT//LFP cell maintained a high areal capacity of 1.51 mAh cm^−2^, retaining 98.7% of its initial capacity, compared to only 60.6% for the Li//LFP cell. Furthermore, rate capability tests over a voltage range of 2.6–4.0 V (Figure 5e) revealed that at a low current density of 0.15 mA cm^−2^ (≈0.1C), both cell types delivered similar capacities of 1.58 mAh cm^−2^. However, at higher current densities, the Li‐SGCNT//LFP cells significantly outperformed the Li//LFP cells, delivering ≈1.50, 1.38, and 1.12 mAh cm^−2^ at 1.5, 3, and 6 mA cm^−2^ (≈1C, 2C, and 4C), respectively. In contrast, the bare Li anode retained only <1 mAh cm^−2^ at 3 mA cm^−2^ and dropped to nearly zero at 6 mA cm^−2^ because of its high overpotential. Additionally, in the charge/discharge profiles (Figure S13, Supporting Information), the Li//LFP cells exhibited higher polarization voltages than the Li‐SGCNT//LFP cells at elevated rates, confirming that the Li‐SGCNT anode effectively enhances Li‐ion transport and reduces the overpotential in full cells. These results represent two significant findings. First, CNTs as a Li host material can achieve high areal capacity (1.5 mAh cm^−2^) and long cycle life (retaining 90% of initial capacity after 800 cycles) while operating at high current densities (1.5 mA cm^−2^) in a full‐cell scenario, which indicates a substantial increase in energy‐storage capacity per unit area. (Note: This degradation may stem from commercial LFP cathodes). Second, this was achieved through the proper selection of CNTs, demonstrating the importance of the starting CNT material. To highlight the performance of our Li‐SGCNT cells, we compared our values (cumulative capacitance, which is the product of capacity, number of cycles, and operational current density) with those reported in the literature (Figure 5f).^[^ ^30^, ^31^, ^32^, ^33^, ^34^, ^35^, ^36^, ^37^, ^38^, ^39^, ^40^, ^41^, ^42^, ^43^ ^]^ We selected these metrics because they are regarded as practical parameters for assessing the performance of Li‐metal batteries. The Li‐SGCNT//LFP full cell exhibited outstanding performance, particularly in terms of cumulative capacity, reaching 1132 mAh cm^−2^ when cycled at 1.5 mA cm^−2^. To the best of our knowledge, these are the highest long‐term cycling parameters reported thus far for full cells featuring a Li‐metal anode and an LFP cathode in a liquid electrolyte. We emphasize that the data used in this comparison were obtained under the same evaluation conditions discussed above, rather than under separate conditions, to maximize the values individually, as is often reported in the literature. While individual optimization can yield higher values for specific metrics, such results do not necessarily reflect meaningful or practical battery performance. Our approach ensures that the comparisons accurately represent the true and consistent performance of our system rather than focusing on artificially maximized values. Finally, we acknowledge that for the results presented here, the commercial cathode serves as the limiting factor in our full battery assembly, and we expect even higher performance with an optimized cathode selection.

The N/P ratio in our full‐cell experiments exceeded 10, which is far higher than that typically used in practical battery systems.^[^ ^44^ ^]^ This excess Li was necessary owing to the use of ≈100‐µm‐thick freestanding CNT films, designed to ensure mechanical stability and to clearly investigate the influence of CNT structure on Li behavior. To maximize the potential of CNT hosts and advance their practical applications, future studies should focus on employing thinner CNT films, thinner Li foils, and higher cathode loadings. Understanding the nature of functional groups is essential for quantitatively isolating the effects of surface chemistry from those of other intrinsic parameters, which is crucial for accurately assessing their impact on the overall performance of CNT‐based Li‐metal anodes. In addition, optimizing the CNT structure through careful selection and controlled functionalization, combined with tailored electrolyte formulations, such as high‐concentration or localized electrolytes,^[^ ^45^ ^]^ can enable more efficient ion transport, promote a more stable SEI layer, reduce parasitic reactions, and stabilize Li deposition under high current densities, ultimately maximizing Li utilization in practical battery systems.

## 3 Conclusion

This study demonstrates that ultrahigh‐capacity and stable Li‐metal anodes can be designed by strategically leveraging the intrinsic structural properties of commercially available CNTs. By systematically evaluating SWCNTs and MWCNTs, we established how variations in diameter, wall number, length, surface chemistry, and assembly structure influence pore characteristics, lithiophilicity, electrolyte permeability, and mechanical robustness. These intrinsic features collectively determine the ability of the CNT host to support uniform Li deposition, enabling exceptional areal capacities (90 mAh cm^−2^) and outstanding cycling stability (over 2000 cycles) during ultrahigh‐current‐density operation (60 mA cm^−2^) in symmetric cells. In full‐cell configurations, the optimized CNT host demonstrated remarkable practical battery performance, with an areal capacity of 1.5 mAh cm^−2^ and stable cycling over 800 cycles at a high current density of 1.5 mA cm^−2^. These values exceeded those reported in the literature. Importantly, these results were obtained without chemical modifications or additional processing steps, underscoring the advantages of informed CNT selection in simplifying the manufacturing process. This work highlights the critical effect of the intrinsic CNT structure on the Li‐metal anode performance, presenting a clear strategy for achieving targeted functionalities in advanced energy‐storage applications through careful material selection rather than extensive chemical treatments.

## 4 Experimental Section

### Preparation of CNT Films

Five types of commercially available CNTs were used without further purification or modification, including three single‐walled CNTs (SGCNT, eDIPS, and Tuball) and two multi‐walled CNTs (JEIO and K‐Nanos). These CNTs were classified by their synthesis methods or manufacturers. SGCNTs were synthesized using the super‐growth method and supplied by Zeon. eDIPS, produced by enhanced direct injection pyrolytic synthesis, were obtained from Meijo Nanocarbon. Tuball, synthesized by a modified floating catalyst method, was purchased from OCSiAl. The multi‐walled JEIO and K‐Nanos, were synthesized by continuous fluidized bed CVD and supplied by JEIO and Kumho Petrochemical, respectively. To fabricate films, 0.1 wt% aqueous dispersions of each CNT type were prepared using high‐speed shear mixing to reduce structural damage. Freestanding CNT films with an areal density of ≈2 mg cm^−^^2^ were obtained by vacuum filtration.

### Structural Characterization

The morphology of CNTs was examined by using a field‐emission SEM (SU8220, Hitachi) and a TEM (EM‐002B, Topcon). For SEM observation, CNT or films were mounted onto carbon tape. For TEM, CNT powders were dispersed in acetone and dropped onto carbon‐coated copper grids. CNT diameter and wall number were statistically evaluated from 50–100 nanotubes. Elemental mapping was conducted using the same SEM system equipped with an EDS detector (Bruker QUANTAX FlatQUAD) using four‐channel silicon drift detectors positioned 2‐3 mm above the sample. EDS was performed at 1 kV and 5 µA, and data were processed using ESPRIT software. The surface area and pore size distribution of CNT films were characterized by nitrogen adsorption isotherms measured at 77 K using a BELSORP‐MAX analyzer (MicrotracBEL). Surface chemistry was examined by FTIR (Vertex 80v, Bruker). CNT films were filtered onto 0.2 µm membranes and transferred to high‐resistivity silicon substrates. Mid‐IR peaks were used to identify functional groups, while far‐IR data were used to estimate CNT length. Raman spectroscopy (Thermo‐Electron, 532 nm laser) was used to assess structural quality. Spectra from more than 10 regions were averaged to determine the G/D ratio as an indicator of crystallinity. Thermal stability was studied by TGA (TG/DTA7300, SII NanoTechnology) under air or nitrogen at 1 °C min^−1^. Weight loss was used to estimate moisture (≈100 °C), functional groups (≈800 °C), and residual content. TPD/MS (TPD‐1‐ATw, MicrotracBEL) was used to analyze the gases released during heating from room temperature to 950 °C at 10 °C min^−1^ under helium. H_2_O, CO, and CO_2_ were monitored, and peak fitting was used to identify contributions from functional groups on CNTs. Gases below 200 °C indicated moisture and labile groups; those from 200–950 °C indicated more stable oxygen‐containing groups.

### Electrochemical Analysis

All battery cells were assembled in an argon‐filled glovebox with oxygen and moisture levels maintained below 1 ppm to prevent degradation of air‐sensitive components. Prior to assembly, CNT films were vacuum‐dried at 200 °C overnight to remove residual moisture. Li plating and stripping behavior was evaluated using symmetric 2032‐type coin cells. Symmetric Li/CNT//CNT/Li cells and control Li//Li cells (16 mm diameter) were fabricated using freshly scraped Li metal foil with a thickness of ≈750 µm (Aldrich). A simple and reproducible assembly procedure was used to ensure good contact between the CNT and Li: the freestanding CNT film was aligned with the Li foil and gently pressed by hand. After the electrolyte injection, a second manual pressing step was applied to remove air gaps and reinforce the interface, while avoiding damage to the CNT film. The electrolyte consisted of 1 M LiPF_6_ dissolved in a 1:1 volume ratio of EC and EMC (Aldrich), with 5 vol% FEC (Aldrich) added as an additive. A commercial polyethylene separator (20 µm thick) was used. Assembled cells were rested overnight to allow full electrolyte infiltration into the CNT films. For full cell assembly, commercial LiFePO_4_ (LFP) cathode sheets (Hohsen) were used. Each cathode had a specific capacity of 1.5 mAh cm^−2^ and consisted of LFP, acetylene black, and poly(vinylidene fluoride) (PVDF) in a weight ratio of 100:5:5. Before use in full cells, the Li/SGCNT composite anodes were preconditioned by Li plating and stripping in symmetric cells (0.5 mA cm^−^^2^, 5 mAh cm^−^^2^, 5 cycles) to remove surface functional groups and form a stable SEI. After an overnight rest to stabilize the interface, Li//LFP and Li–SGCNT//LFP full cells were cycled between 2.5 and 4.0 V at current densities ranging from 0.15 to 6 mA cm^−^^2^. Galvanostatic charge–discharge testing was performed using a HJ1001SD8 battery tester (Hokuto Denko). EIS was carried out using a VMP3 workstation (BioLogic) with a 5 mV AC perturbation over a frequency range of 10 mHz to 500 kHz to evaluate interfacial resistance and overall cell impedance.

## Conflict of Interest

The authors declare no conflict of interest.

## Supporting information



Supporting Information

## Data Availability

The data that support the findings of this study are available from the corresponding author upon reasonable request.
