# Supplementary material for: Designing High‐Rate and High‐Capacity Lithium Metal Anodes: Unveiling Critical Role of Carbon Nanotube Structure
Source: Small. 2025 Aug 7;21(38):e03161. doi: 10.1002/smll.202503161 (PMC12462607; doi:10.1002/smll.202503161)
Supplement: Supplementary file 1 — Supporting Information [file SMLL-21-e03161-s001.docx]

Supporting Information

Designing High-Rate and High-Capacity Lithium Metal Anodes: Unveiling Critical Role of Carbon Nanotube Structure

*Ying Zhou,^*^ Tomoko Yamagishi, Kazufumi Kobashi, Don N. Futaba, Takeo Yamada, Kenji Hata*

Y. Zhou,^*^ K. Kobashi, D.N. Futaba, T. Yamada, K. Hata

Nanocarbon Material Research Institute,

National Institute of Advanced Industrial Science and Technology,

1-1-1 Higashi, Tsukuba, Ibaraki 305-8565, Japan.

E-mail: y-shuu@aist.go.jp

T. Yamagishi

Zeon Corporation,

1-2-1 Yako Kawasaki-ku, Kawasaki, Kanagawa 210-9507, Japan


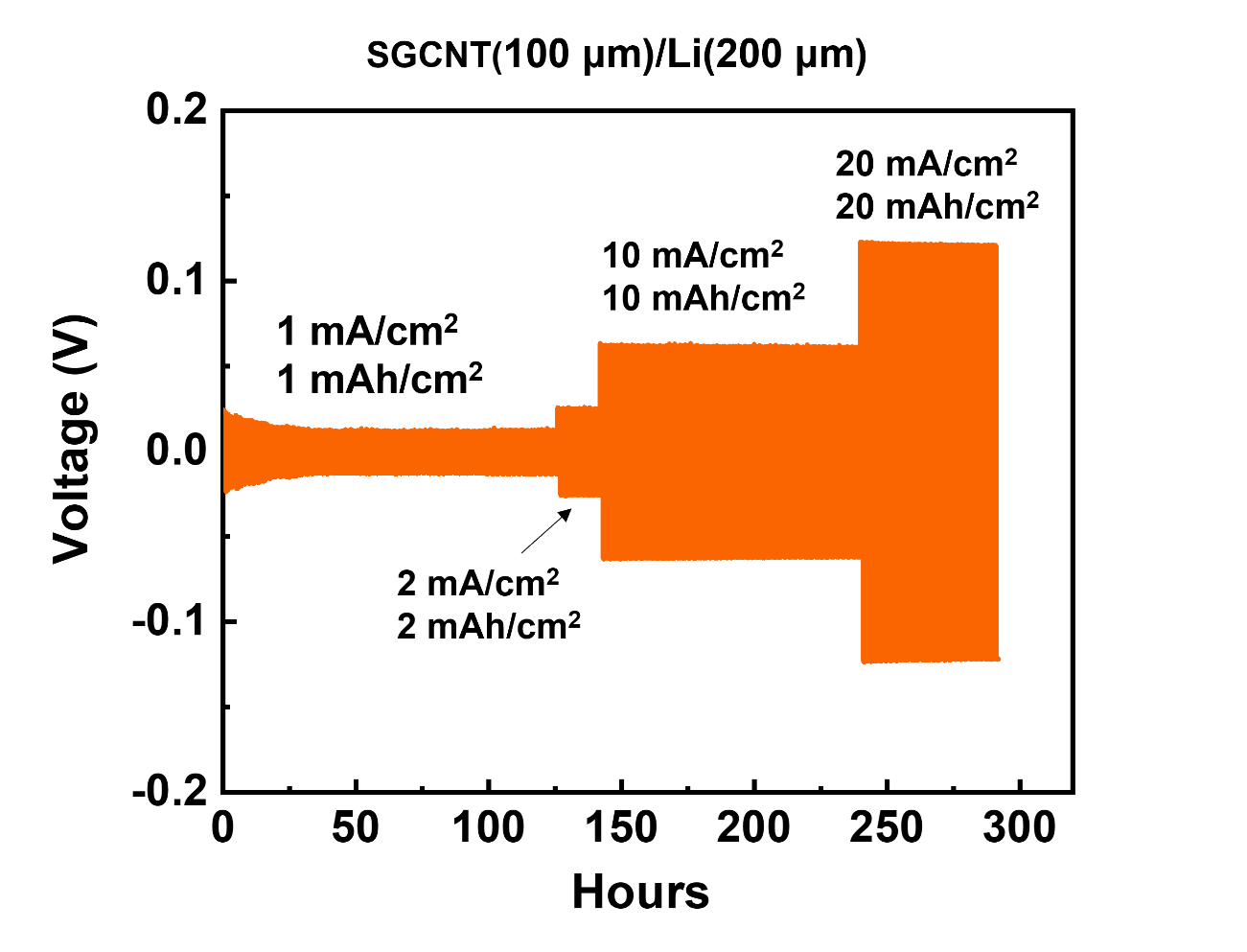


Figure S1 Galvanostatic cycling profiles of Li-SGCNT//SGCNT-Li symmetric cells using 200 μm thick Li.


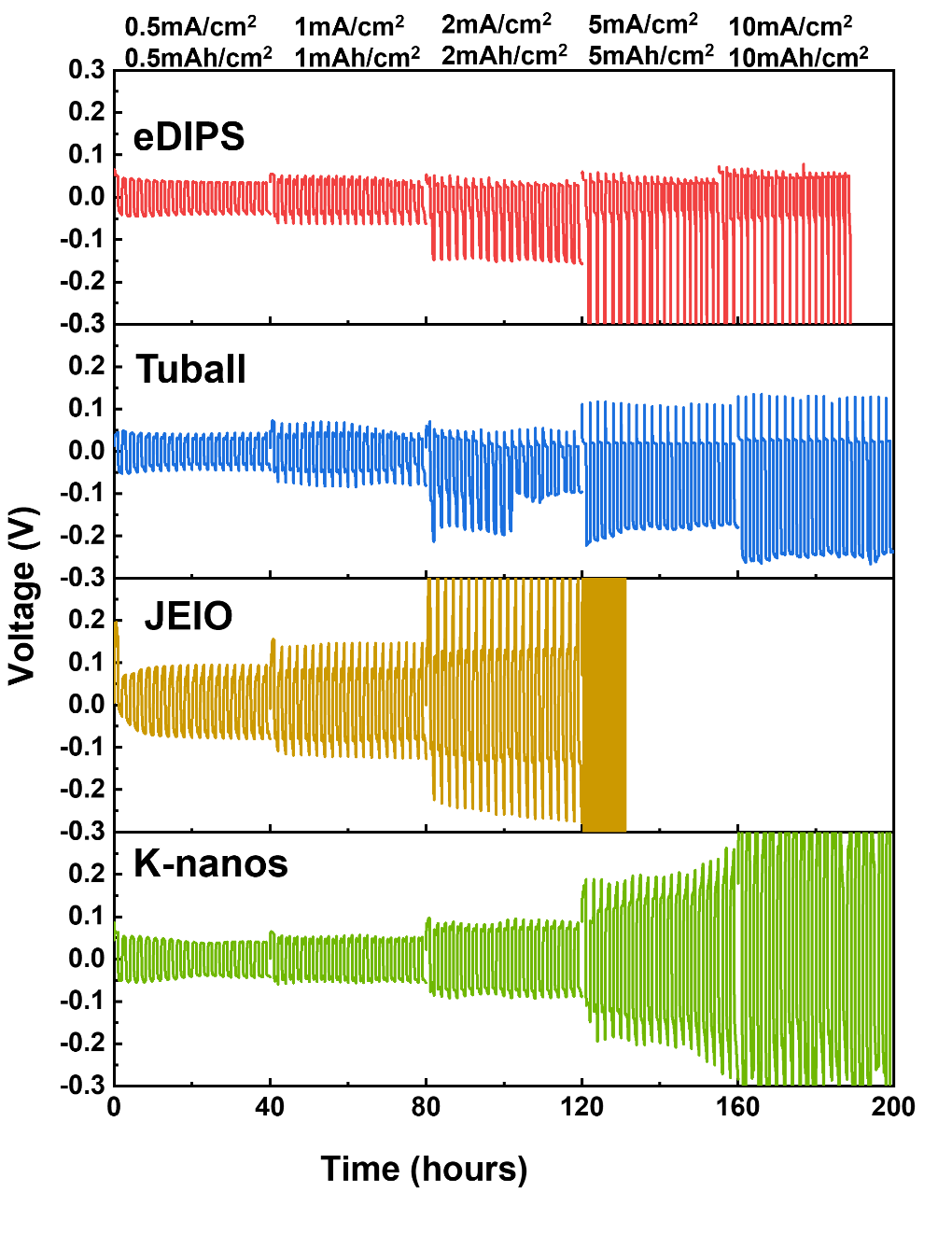


Figure S2. Galvanostatic cycling profiles of Li-CNT//CNT-Li symmetric cells using various commercial CNTs, including eDIPS, Tuball, JEIO, and K-Nanos. The results indicate that these symmetric cells exhibited noticeable degradation at current densities above 2 mA/cm².


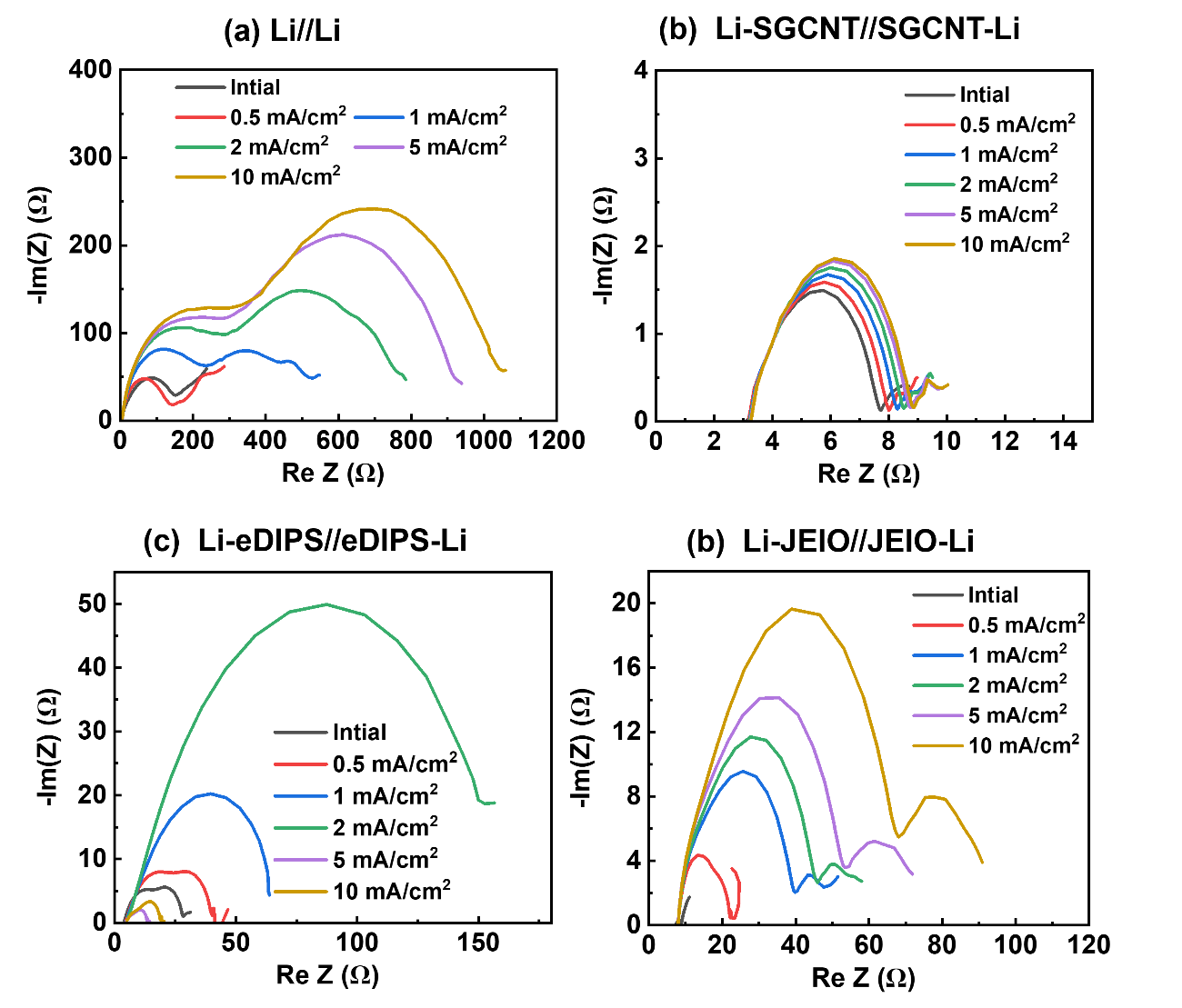


Figure S3 Electrochemical impedance spectroscopy (EIS) of (a) Li//Li, (b) Li-SGCNT//SGCNT-Li, (c) Li-eDIPS//eDIPS-Li and (d) Li-JEIO//JEIO-Li.


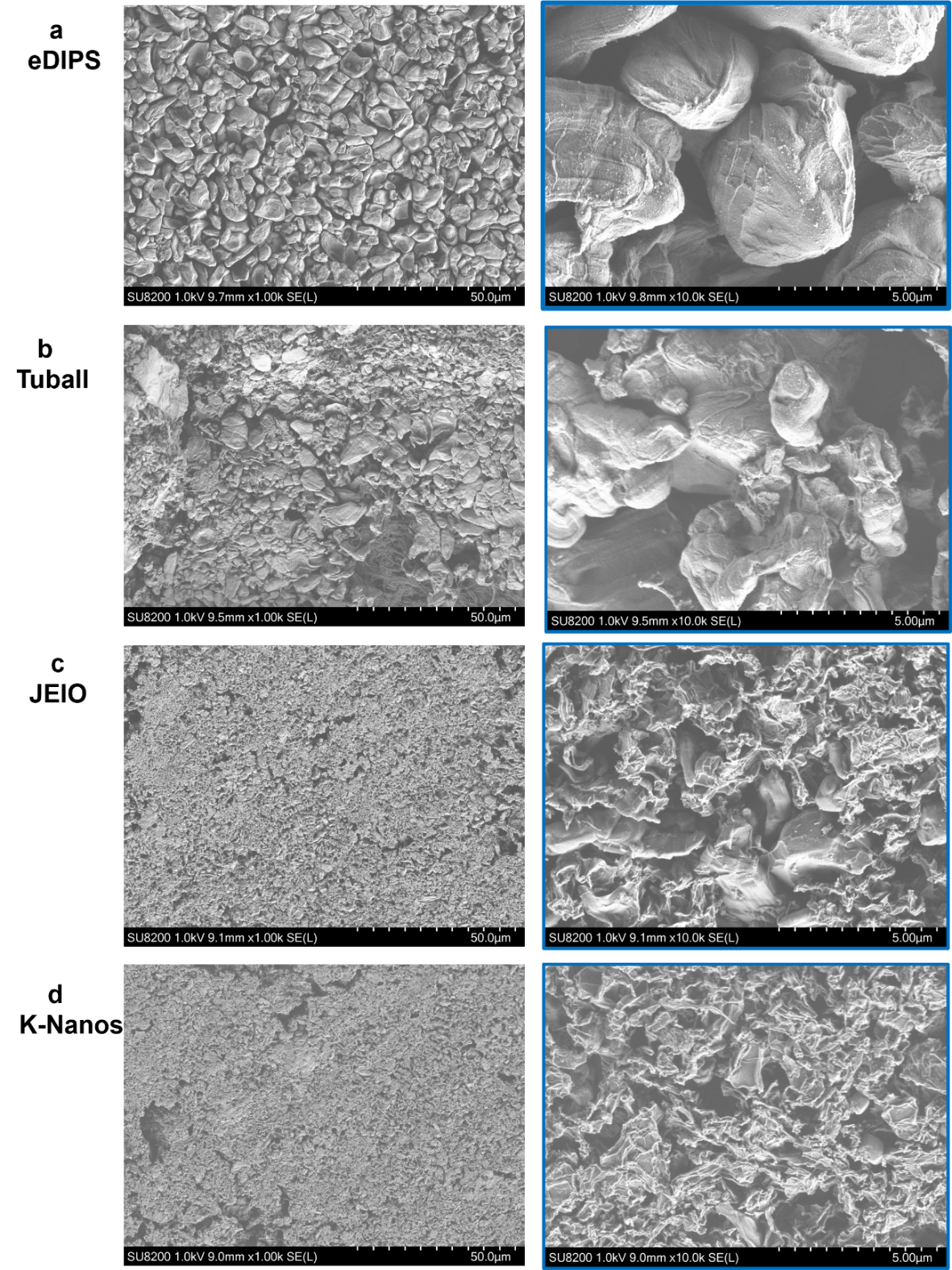


Figure S4. SEM images of (a) eDIPS, (b) Tuball, (c) JEIO and (d) K-Nanos after cycling tests shown in Figure S1. The results indicate significant crystalline Li growth on eDIPS and Tuball, while small dead Li agglomerates were observed on JEIO and K-Nanos.


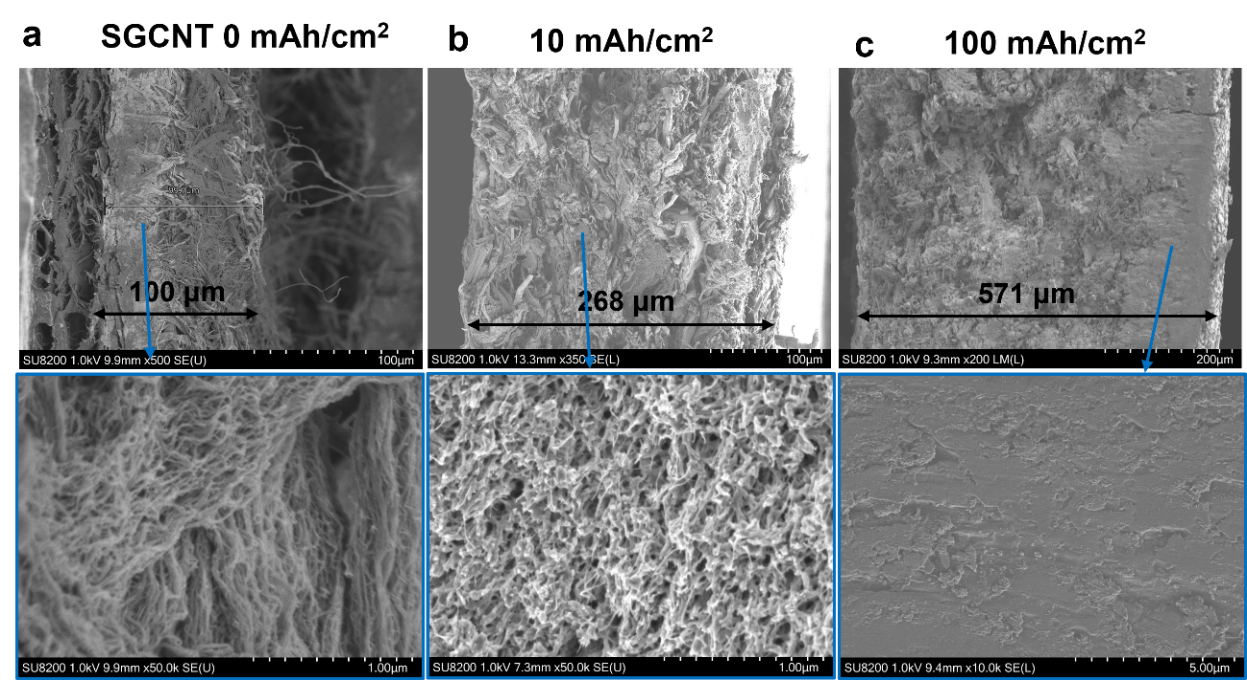


Figure S5. Cross-sectional SEM images of SGCNT films (a) before Li plating and after plating at (b) 10 mAh/cm² and (c) 100 mAh/cm². The results clearly show that the pore structure directs Li deposition, increasing the SGCNT film thickness by more than three times to accommodate Li, demonstrating its effectiveness as a Li host. Ultimately, a dense Li layer forms on the SGCNT surface, indicating that the SGCNT host efficiently guides Li growth, enabling ultrahigh capacity. Moreover, the SGCNT film thickness after Li plating at 10 mAh/cm² is twice that of the SGCNT film after cycling at 10 mAh/cm² (as shown in Figure 2), possibly suggesting structural adaptability that enables stable Li plating/stripping at high capacities.


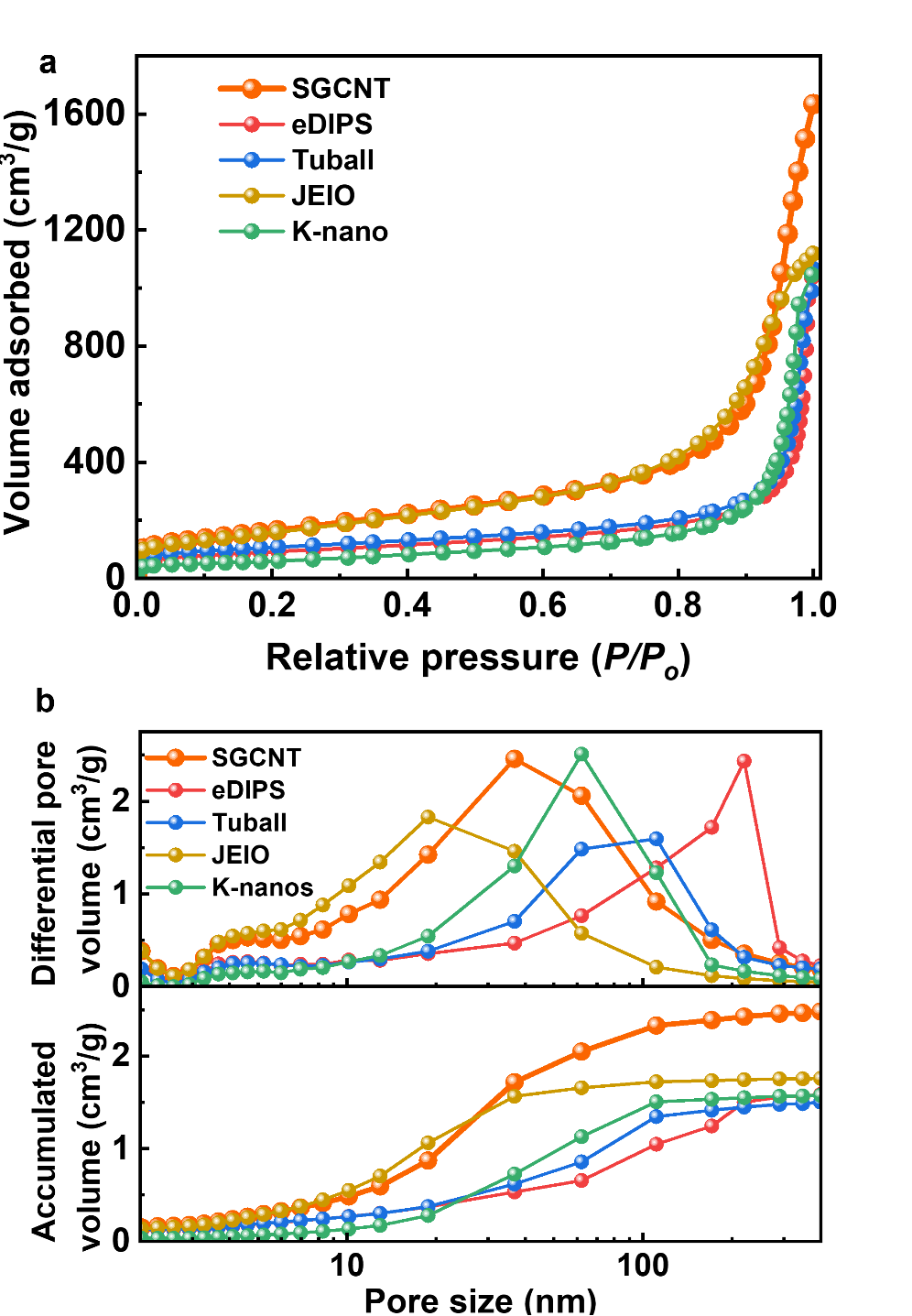


Figure S6. (a) N₂ adsorption isotherms and (b) pore size distribution of CNT films. The pore size distribution of CNTs is consistent with the pore structures observed in TEM images shown in Supplementary Figure 5. SGCNT exhibits dominant pore structures in the range of 30–70 nm.


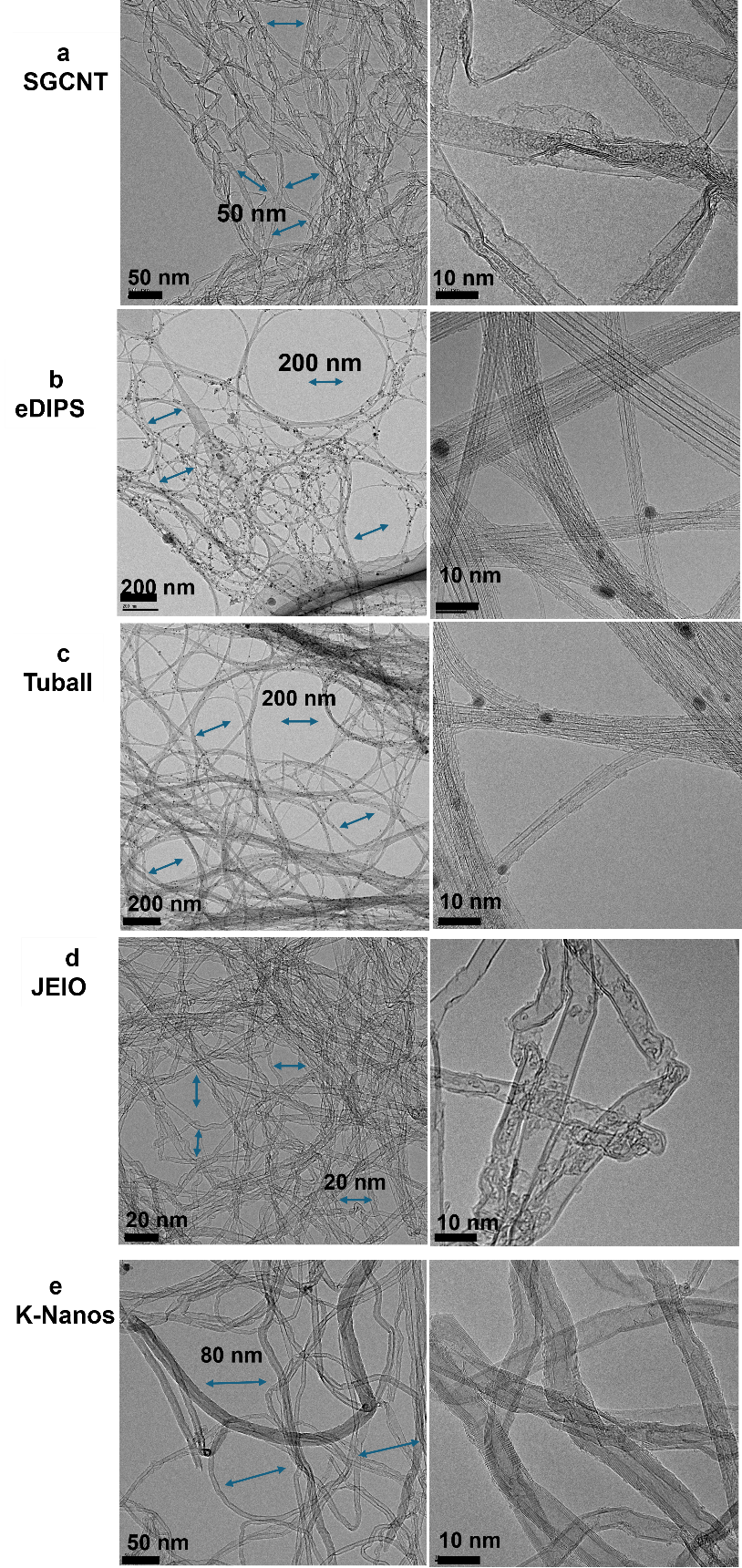


Figure S7. TEM images of (a) SGCNT, (b) eDIPS, (c) Tuball, (d) JEIO, and (e) K-Nanos, illustrating variations in diameter, wall number, impurity content, and pore structure among different CNTs.


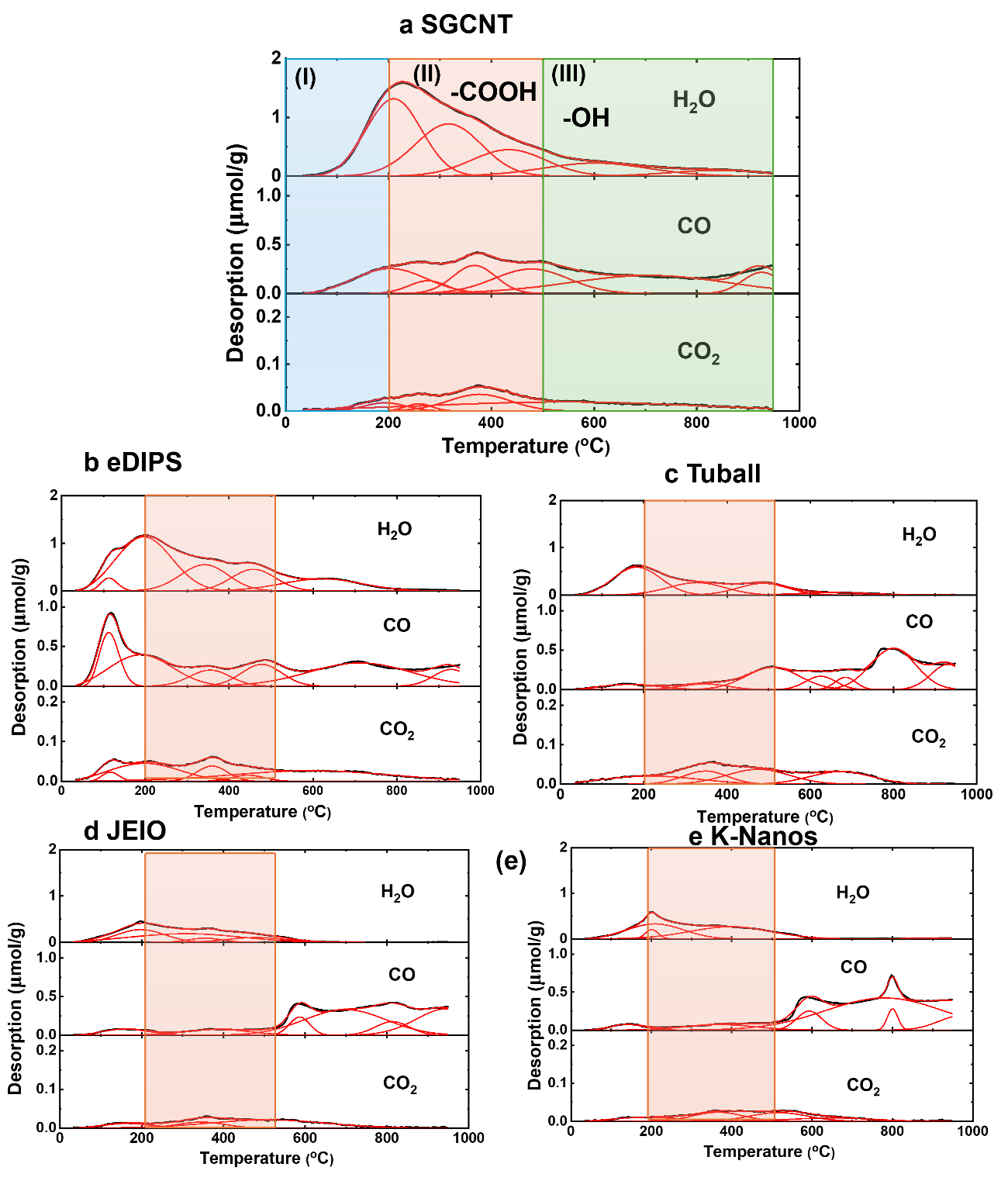


Figure S8. TPD-MS analysis of (a) SGCNT, (b) eDIPS, (c) Tuball, (d) JEIO, and (e) K-Nanos, illustrating variations in functional groups among CNTs. ^[1]^ Notably, the high desorption observed in eDIPS CNTs may be attributed to gas molecules trapped within the CNT bundles rather than the presence of functional groups. It has been reported that high-temperature treatment in vacuum can effectively remove these trapped gases. ^[2]^ This is further confirmed by Raman, FTIR, and XPS spectra, which indicate that eDIPS exhibits high crystallinity with minimal functional groups.


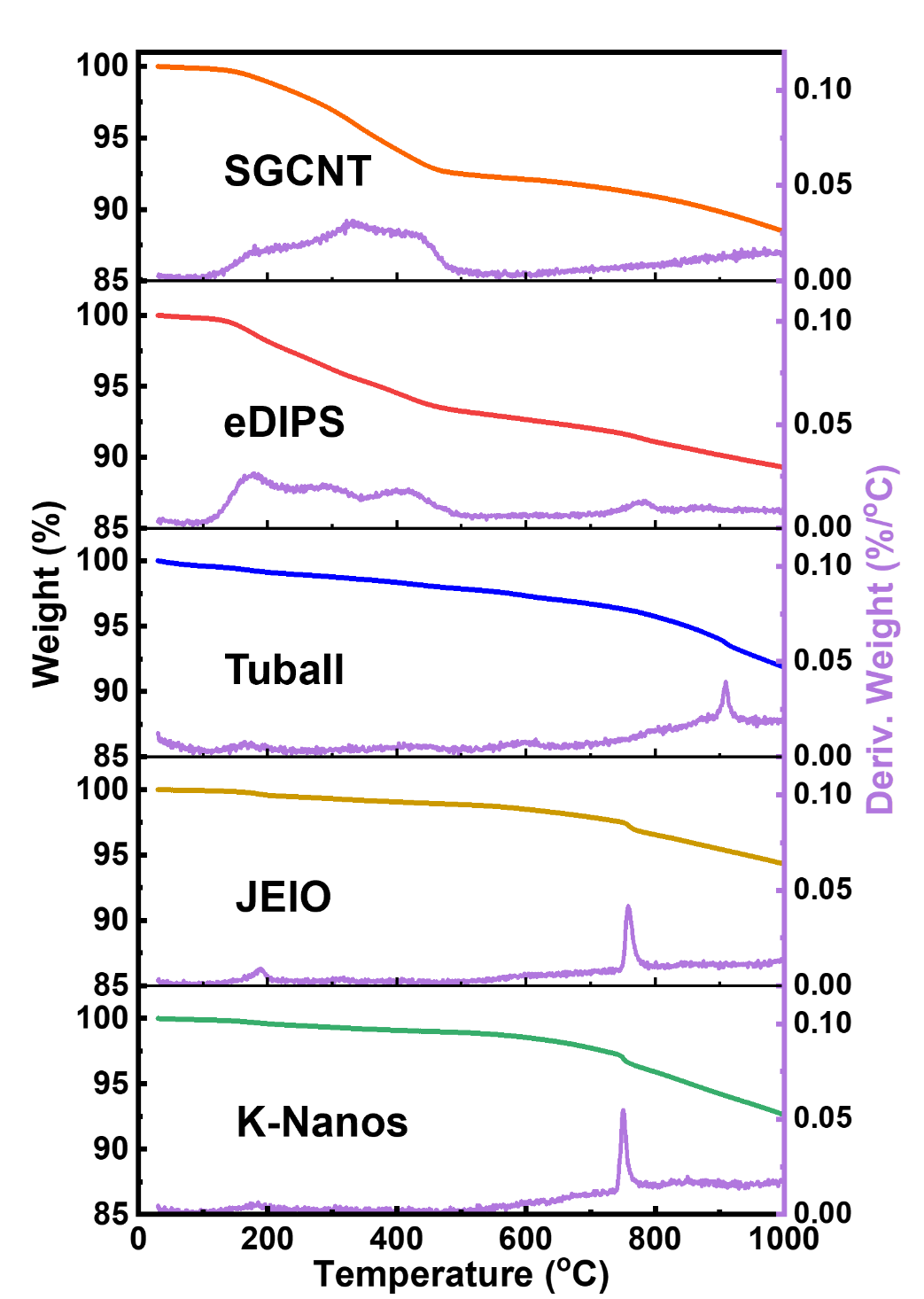


Figure S9. TGA results of the CNTs. The results align with TPD-MS. the weight loss observed in eDIPS is likely due to trapped gas rather than the presence of functional groups.


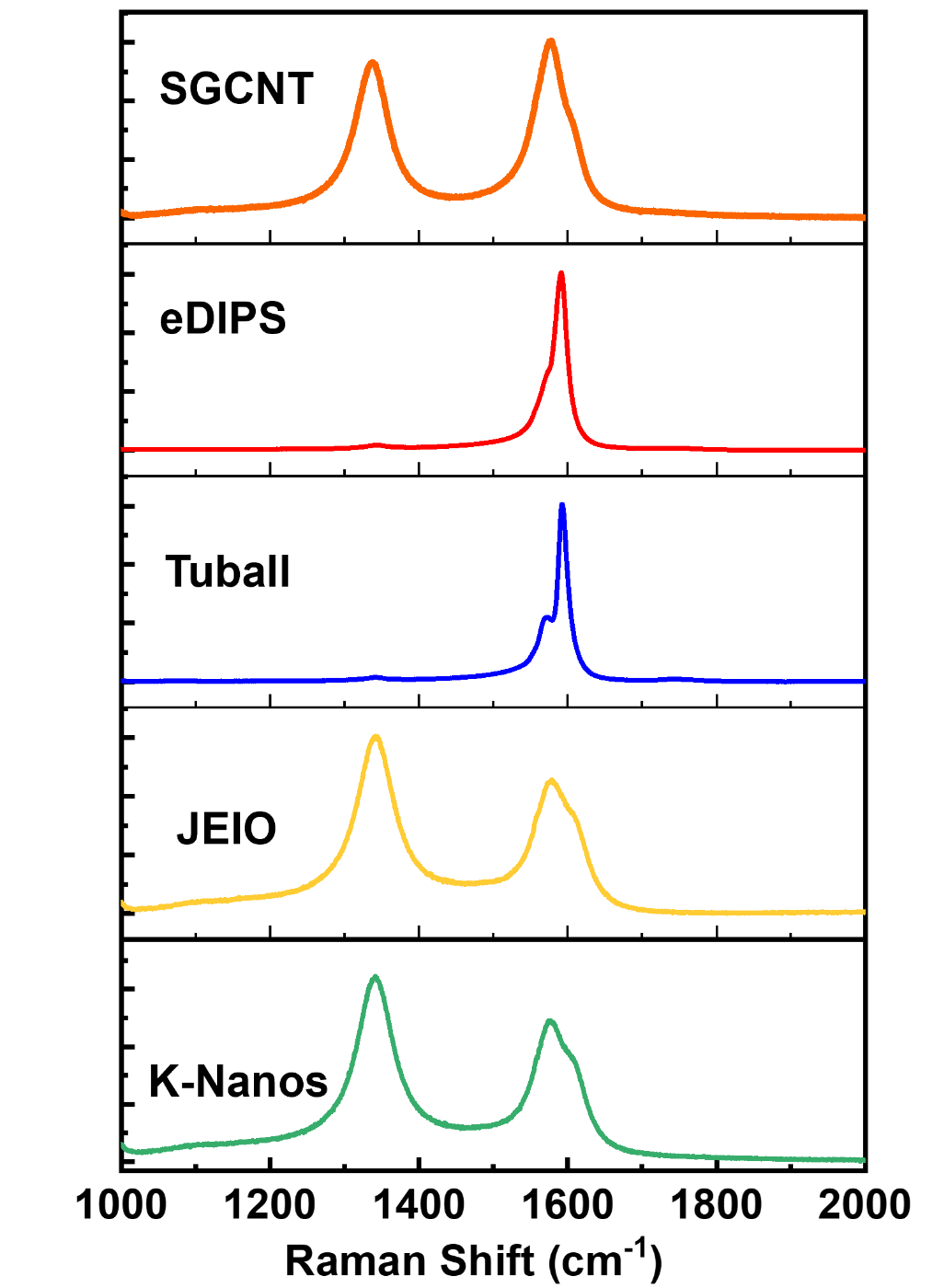


Figure S10. Raman spectra of CNTs, showing that eDIPS and Tuball exhibit higher crystallinity with fewer structural defects.


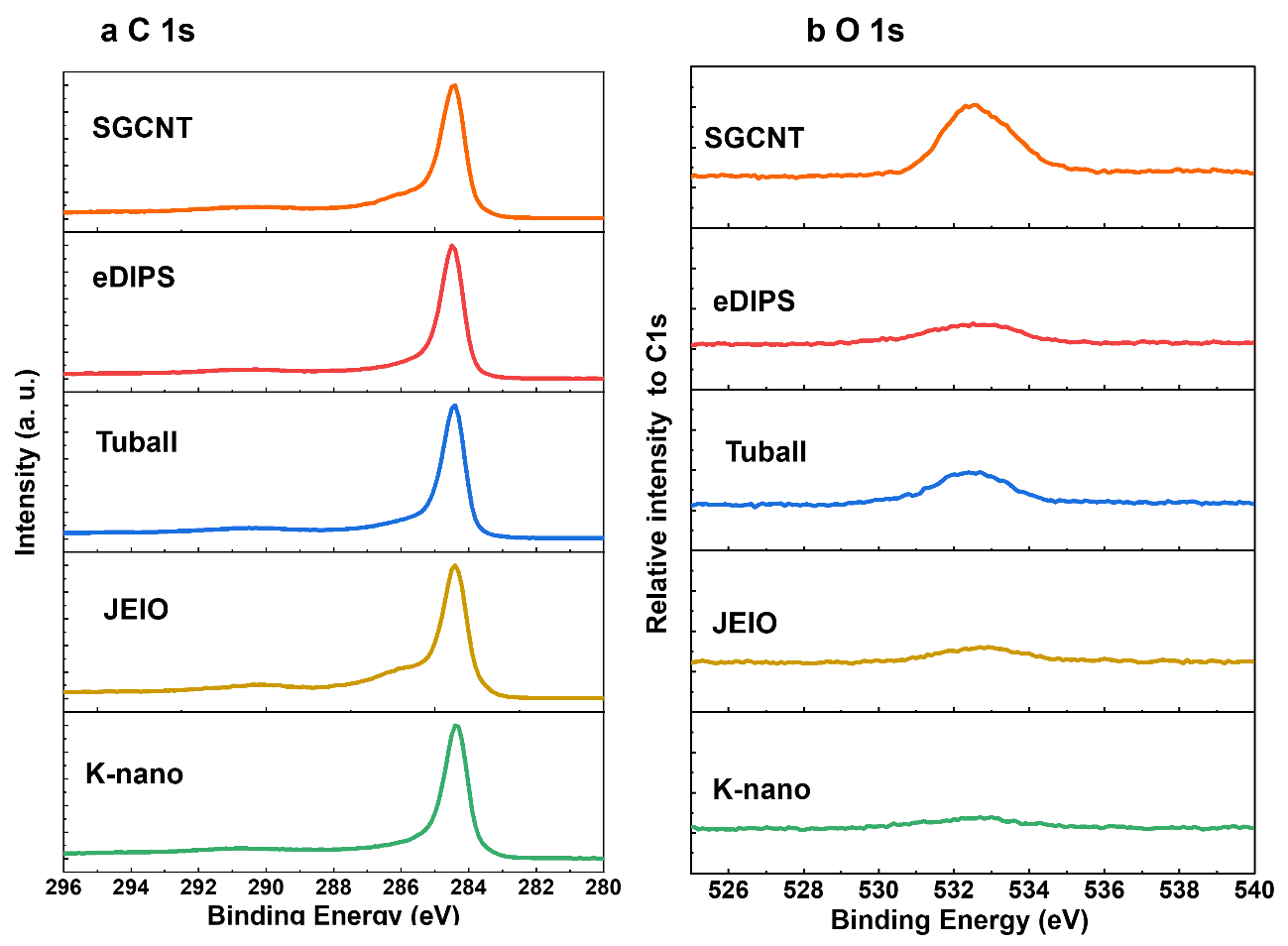


Figure S11. XPS spectra of CNTs: (a) C 1s and (b) O 1s, normalized to the C 1s peak at ~284.8 eV. The highest O 1s signal in SGCNTs suggests that its structural defects are primarily attributed to the oxygen-containing functional groups. Notably, although JEIO and K-Nanos exhibit significant structural defects in Raman, they do not show a substantial presence of oxygen-containing functional groups.


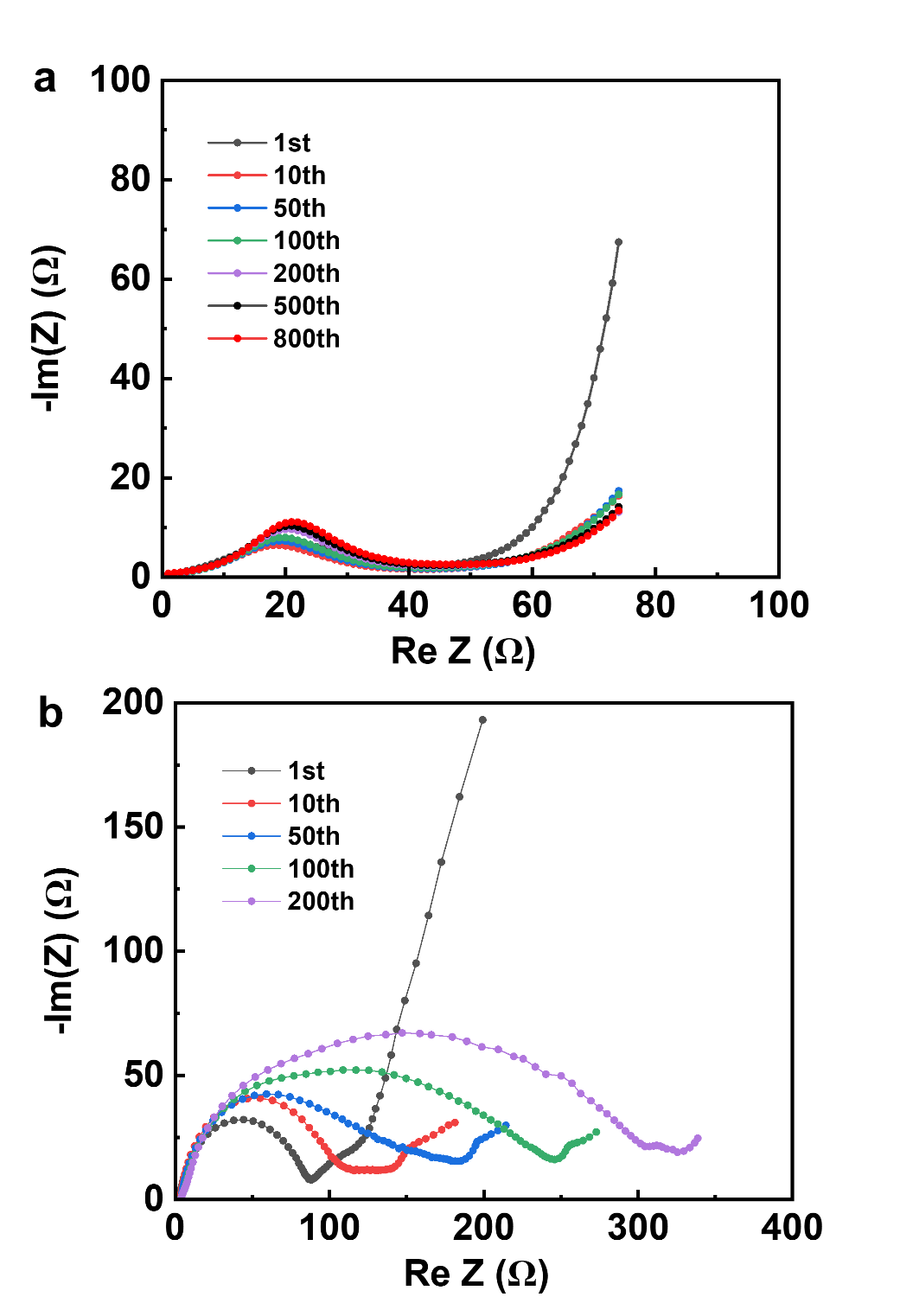


Figure S12 Electrochemical impedance spectroscopy (EIS) of (a) Li-SGCNT//LFP and (b) Li//LFP full cells at 1.5 mA/cm^2^. The Li-SGCNT//LFP cell exhibited smaller and stable impedance after 800 cycles, consistent with the results in Fig. 5a. In contrast, the increased impedance observed in the Li//LFP cell indicates that dendrite growth leads to higher resistance and, consequently, an increased overpotential.


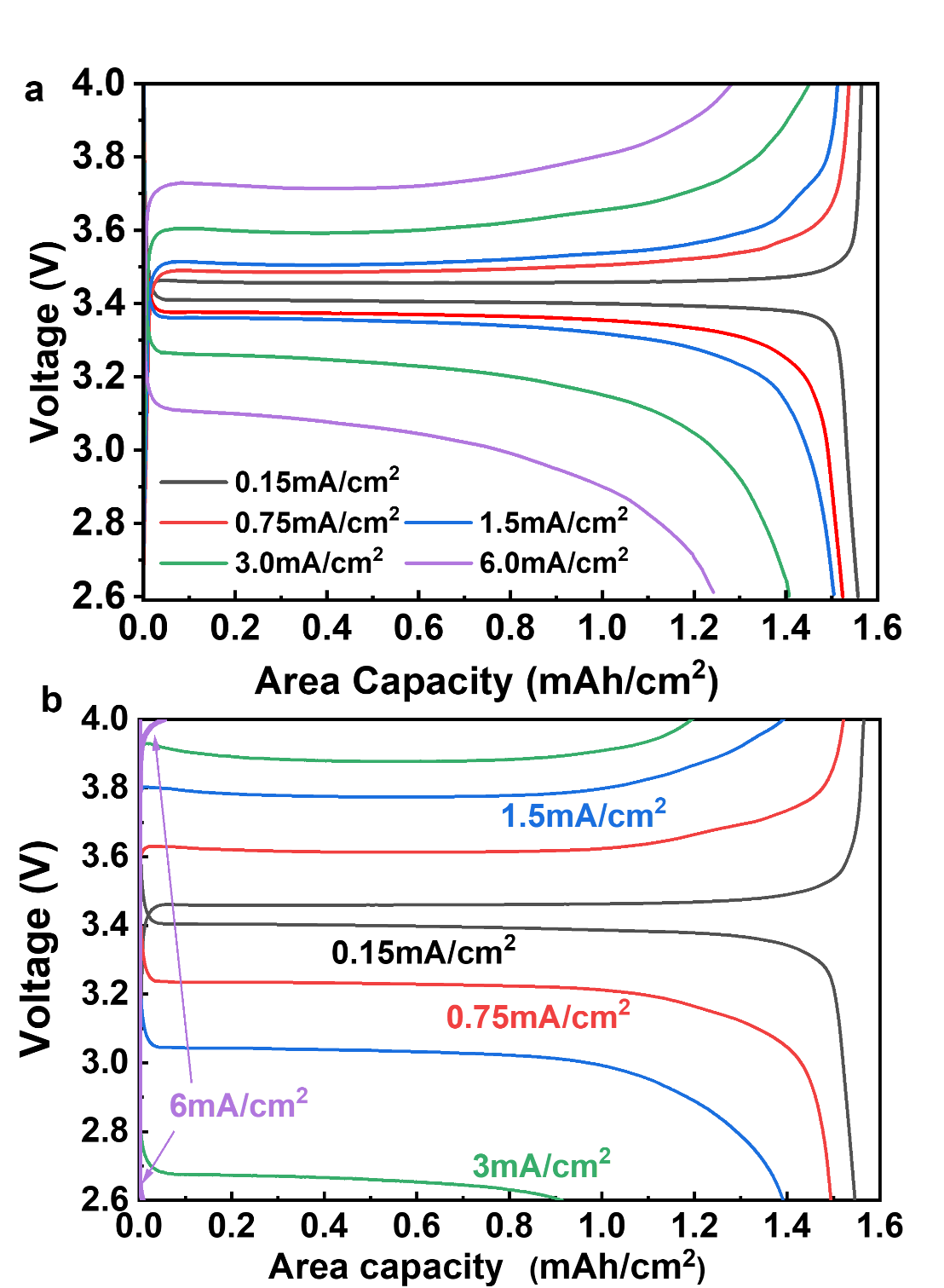


Figure S13. Galvanostatic voltage profiles of (a) Li-SGCNT//LFP and (b) Li//LFP full cells at various current densities ranging from 0.15 to 6 mA/cm². The performance of the full cells is influenced by both the Li anode and the LFP cathode. The Li-SGCNT anode demonstrates high potential for high-rate and high-energy-density Li-metal batteries.

Table S1 Summary of symmetric cell tests of Li metal anodes operated at high current density and/or high area capacity and/or high cycle number.

| Method | Current density  (mA/cm^2^) | Capacity  (mAh/cm^2^) | Cycle number | Accumulative cycled capacity  (mAh/cm^2^) | ref. |
| --- | --- | --- | --- | --- | --- |
| Li-SGCNT | 30 | 120 | 222 | 26640 | This work |
|  | 40 | 120 | 251 | 30120 |  |
|  | 60 | 90 | 2004 | 180360 |  |
| LiSi alloy | 25 | 100 | 2 | 200 | 5 |
| CNT with infused Li | 40 | 2 | 20000 | 40000 | 10 |
| N-Doped carbon nanospheres | 64 | 1 | 50 | 50 | 6 |
| Functionalized graphene | 60 | 60 | 550 | 33000 | 7 |
| Modified carbon | 10 | 30 | 1080 | 32400 | 14 |
| CNT with infused Li | 10 | 10 | 1000 | 10000 | 15 |
| Organic layer | 10 | 10 | 1400 | 14000 | 16 |
| Functionalized CNT | 15 | 15 | 300 | 4500 | 17 |
| Inorganic-dominant SEI | 30 | 30 | 4000 | 120000 | 18 |
| 3D carbon with functionalized graphene | 40 | 40 | 260 | 10400 | 19 |
| Artificial SEI | 20 | 20 | 500 | 10000 | 20 |
| ZnO/CNT | 5 | 20 | 50 | 1000 | 21 |

Table S2 The properties of CNT raw powders and films

| CNT^a^ | Raw powders | | | Film | | | | | | | | |
| --- | --- | --- | --- | --- | --- | --- | --- | --- | --- | --- | --- | --- |
|  | Diameter  (nm) ^b^ | Wall No.^b^ | Effective length (nm)^c^ | G/D  ratio | BET  SSA  (m^2^/g) | Pore size  (nm) ^d^ | Weight loss by TGA  (wt%) | -COOH (wt%)^e^ | FG  (wt%)^f^ | Conductivity  (S/cm) | Porosity  (%)^g^ | Film thickness  (μm) |
| SGCNT | 4.8 | 1.5 | 55 | 1.2 | 600 | 45 ± 20 | 11.5 | 4.1 | 5.9 | 40 ± 10 | 90~95 | 100~120 |
| eDIPS | 1.5 | 1.1 | 3000 | 65 | 330 | 200 ± 50 | 10.7 | 2.3^h^ | 4.9^h^ | 350 ± 150 | 70~75 | 50~60 |
| Tuball | 1.6 | 1.0 | 1072 | 60 | 376 | 110 ± 40 | 8.2 | 1.4 | 3.2 | 350 ± 150 | 70~75 | 50~60 |
| JEIO | 4.3 | 2.4 | - | 0.8 | 585 | 25 ± 15 | 5.7 | 1.3 | 3.0 | 10 ± 5 | 85~90 | 80~100 |
| K-Nanos | 9.1 | 6.2 | - | 0.8 | 212 | 70 ± 25 | 7.4 | 1.1 | 2.7 | 10 ± 5 | 85~90 | 80~100 |

(a) The commercial CNTs used in this study are SGCNT (Zeonano), eDIPS (EC1.5), Tuball (01RW02), JEIO (JC142), and K-Nanos (K-Nanos-100p).
(b) The CNT diameter and wall number were estimated by analyzing over 100 tubes in TEM images.
(c) The effective CNT length was estimated based on the plasmon resonance peak in the far-IR range; no significant peak was observed for JEIO and K-Nanos tubes.
(d) The pore size distribution was determined using the Barrett-Joyner-Halenda (BJH) method from N₂ adsorption isotherms, revealing distinct characteristics.
(e) Functional groups, primarily –COOH, were quantified by TPD-MS within the 200–500 °C range.
(f) The total functional group concentration was estimated by TPD-MS within the 200–900 °C temperature range.
(g) The film porosity was estimated by comparing the measured film density (weight per volume) with the theoretical density of close-packed CNT bundles (1.5 g/cm³).
(h) The concentration of functional groups in eDIPS may be overestimated, possibly due to the presence of trapped gas molecules.^S2^

Table S3 Summary of liquid-electrolyte-based full cell tests using Li metal anodes operated at high current density and/or high area capacity and/or high cycle number.

| Method | Cathode | Current density  (mA/cm^2^) | Capacity  (mAh/cm^2^) | Cycle number | Accumulative cycled capacity ^a^  (mAh/cm^2^) | Ref. |
| --- | --- | --- | --- | --- | --- | --- |
| Li-SGCNT | LFP | 1.5 | 1.5 | 800 | 1132 | This work |
|  |  | 0.75 | 1.5 | 200 | 356 |  |
| Polymer/inorganic coating | LFP | 1 | 1 | 500 | 500 | 30 |
| 3D framework | LFP | 1.3 | 1.3 | 350 | 455 | 31 |
| Lithophilic membrane | LFP | 1.1 | 0.55 | 1000 | 550 | 32 |
| Organic/inorganic interface | LFP | 1.7 | 1.4 | 320 | 448 | 33 |
| Li_2_S/Li_2_Se layer | LFP | 0.8 | 0.8 | 460 | 368 | 34 |
| CoSe/carbon host | NMC | 0.16 | 0.8 | 300 | 240 | 35 |
| GF/LiF coating | LFP | 2 | 1 | 300 | 300 | 36 |
| CNT with infused Li | LFP | 1 | 1 | 100 | 100 | 37 |
| Polymer coating | NMC | 1.4 | 1.4 | 200 | 280 | 38 |
| Artificial SEI | LFP | 1.7 | 1.7 | 200 | 340 | 39 |
| COF host | LFP | 0.6 | 0.6 | 650 | 390 | 40 |
| MgH_2_/C_3_N_4_ host | LFP | 1.7 | 1.5 | 450 | 675 | 41 |
| PVA coating | LFP | 0.45 | 1.4 | 400 | 560 | 42 |
| Electrolyte optimization | LFP | 2.5 | 0.25 | 1200 | 300 | 43 |

^a^The cumulative cycled capacity represents the total discharge capacity over all cycles in this work, whereas it is typically estimated by multiplying the first-cycle discharge capacity by the number of cycles for the literature.

References.

[1] R. P. Rocha, M. F. R. Pereira, J. L. Figueiredo, *Catalysis Today* 2023, *418*

[2] K. Kobashi, S. Yamazaki, K. Michishio, H. Nakajima, S. Muroga, T. Morimoto, N. Oshima, T. Okazaki, *Carbon* 2023, *203*, 785-800
